# Development of a novel konjac glucomannan/soy protein isolate/fatty acid composite film: Insights into the structure, properties and interaction mechanism

**DOI:** 10.1371/journal.pone.0340257

**Published:** 2026-02-09

**Authors:** Xiumei Wang, Qingyu Song, Lulu Li, Wenting Lin, Xiaoxu Zhao, Jie Pang

**Affiliations:** 1 College of Environmental and Biological Engineering, Putian University, Putian, China; 2 Fujian Provincial Key Laboratory of Ecological Impacts and Treatment Technologies for Emerging Contaminants, Putian University, Putian, China; 3 Key Laboratory of Ecological Environment and Information Atlas (Fujian Provincial University), Putian University, Putian, China; 4 College of Food Science, Fujian Agriculture and Forestry University, Fuzhou, China; Henan Institute of Science and Technology, CHINA

## Abstract

The preparation, structural characterization and properties analysis of novel konjac glucomannan (KGM)/soy protein isolate (SPI)/fatty acid (KS-FA) films were carried out in this paper. KS-FA films exhibited higher crystallinities than KS film. Moreover, incorporating fatty acids significantly affected the surface roughness and morphologies of KS film. KS-FA films had significantly (p < 0.05) higher water contact angle values than KS film. The rheological properties of the film-forming solution, thermal stabilities, mechanical, water vapor barrier and water resistance properties of KS film were also improved after incorporating appropriate concentrations of fatty acids. There were a 21.3-percent increase of glass transition temperature value, a 17-percent increase of water contact angle value, a 14.5-percent decrease of water solubility value, a 43-percent decrease of water vapor permeability value, a 1.08-fold increase of tensile strength value, and a 1.52-fold increase of elongation at break value in KS film with 0.7% lauric acid when compared with those of KS film. Furthermore, molecular docking and fourier transform infrared spectroscopy analyses suggested that KS-FA films were formed through hydrogen bonds and hydrophobic interactions. These findings highlight fatty acid-modified KGM/SPI films as promising biodegradable food packaging materials.

## 1. Introduction

Nowadays, more and more non-degradable petroleum-based packaging materials are replaced by the environmentally friendly biodegradable films due to their serious white pollution and food safety problems [1–3]. Moreover, many studies have showed that various natural biodegradable polymers like polysaccharides, proteins, and lipids have been widely employed to prepare food packaging films [4–8]. Among them, soy protein isolate (SPI) and konjac glucomannan (KGM) have received increasing attentions because of their excellent properties such as non-toxicity, good biodegradability, film-forming ability, and biocompatibility [9–11]. However, pure KGM and SPI films still have their own unfavorable properties like low mechanical properties, poor thermal stability and water resistance [12,13]. Therefore, preparation of the composite films by combining two or more biopolymers is useful to achieve enhanced properties [14–16].

Considerable studies have indicated that blending KGM with SPI could promote synergistic effects [17–20]. Moreover, it was reported that the composite films after mixing KGM with SPI exhibited relatively higher thermostability, mechanical properties and water vapor barrier properties than the corresponding pure films [21–23]. However, the water vapor barrier properties of KGM/SPI (KS) film still can’t meet the current requirements of food packaging films and need to be further enhanced. Recent studies have suggested that fatty acids such as stearic acid (SA), palmitic acid, lauric acid (LA) are good candidates for the development of food packaging films with better water vapor barrier properties because of their high hydrophobicity [24–26]. Therefore, incorporating fatty acids into KS film may be an effective strategy to improve the water vapor barrier performances.

Although there are numerous studies about the biodegradable films containing fatty acids [27,28], it is still unclear whether the influences of different fatty acids on the properties and structure of different food packaging films are similar. Moreover, no literatures have been reported on the preparation of KGM/SPI/fatty acid (KS-FA) films and their related properties. Considering that proteins are prone to denaturation at excessive temperatures and unsaturated fatty acids are easily oxidized, saturated fatty acids are chosen in this paper [25,29]. Among them, SA and LA are commonly employed as hydrophobic constituents in the preparation of food packaging films because of their low cost, rich source, good biodegradability, mild melting temperatures (c.a, 72°C for SA and 44°C for LA), and classification as generally recognized as safe (GRAS) by the Food and Drug Administration (FDA) [25,28,29]. Therefore, the main objective of this paper was to evaluate the effects of SA and LA on the structure and properties of KGM/SPI film. Firstly, the preparation of KS films with different concentrations of SA and LA was carried out using a solution casting method. Then, the rheological properties of all the film-forming solutions were studied. Subsequently, the structure of KS-FA films was characterized by fourier transform infrared spectroscopy (FTIR), X-ray diffractometer (XRD), differential scanning calorimetry (DSC), scanning electron microscopy (SEM), and their mechanical properties, water vapor permeability (WVP), water solubility (WS) and water contact angle (WCA) were also investigated. Finally, molecular docking was conducted to discuss the possible formation mechanism of KS-FA films.

## 2. Materials and methods

### 2.1. Materials

Anhydrous calcium chloride, konjac glucomannan (≥95% purity) and stearic acid (98%) were bought from Shanghai Aladdin Biochemical Technology Co., Ltd. (Shanghai, China). Glycerol and anhydrous ethanol were supplied by China National Pharmaceutical Group Chemical Reagent Co., Ltd. (Shanghai, China). Lauric acid (98%) and soy protein isolate were purchased from Shanghai Maclean’s Biochemical Technology Co., Ltd. (Shanghai, China). All other chemical reagents used were of analytical grade unless otherwise specified.

### 2.2. Preparation of KGM/SPI/fatty acid films

0.75 g KGM was mixed with 100 mL of distilled water and 0.05 g glycerol at 70°C with continuous stirring for 1 h to prepare pure KGM film solution. 5 g SPI and 1.5 g glycerol were added to 100 mL of distilled water at 70°C with continuous stirring for 20 min to form pure SPI film solution. Then, these two pure film solutions were mixed together at a weight ratio of 15:1 and continuously stirred at 70°C for 20 min to obtain KGM/SPI film solution. After that, LA (0.5, 0.6, 0.7, 0.8 and 0.9%) and 0.8% SA-ethanol solution (0.02, 0.04, 0.06, 0.08 and 0.10%) were loaded into KGM/SPI film solution at 70°C with continuous stirring for 20 min to prepare KGM/SPI/LA and KGM/SPI/SA film solutions, respectively. Subsequently, glass Petri dishes containing various single and composite film solutions were dried at 60°C for 6 and 8 h, respectively. Finally, various dried films after peeling off were balanced in a constant temperature and humidity box with a temperature of 25°C and a relative humidity of 50% before analysis. The obtained films were sequentially named as KGM, SPI, KS, KSL_5_, KSL_6_, KSL_7_, KSL_8_, KSL_9_, KSS_2_, KSS_4_, KSS_6_, KSS_8_, KSS_10_, respectively. All fatty-acid concentrations on a dry-mass basis (g/100 g total solids) and the total dry mass per cast fatty-acid dispersion are shown in S1 Table.

### 2.3. Rheological properties of various film solutions

The rheological properties of various film solutions were analyzed by a Hack Rheometer (Viscotester iQ, Hack Corporation, Germany) at 25°C for 30 s. The speed of FL 22 4B/SS-01160184 rotor was 70 r/s. During this period, data point was collected every two seconds.

### 2.4. FTIR analysis of various films

Prior to analysis, various films were cropped to small square piece with the size of 2 cm × 2 cm. Then, a TENOSOR27 FTIR spectrometer (Burker Corporation, Germany) was employed to measure the FTIR spectra of various films in the wavenumber range of 400−4000 cm^-1^. The resolution was 4 cm^-1^ with an average of 32 scans.

### 2.5. XRD analysis of various films

Before the measurement, various films were cut into small disks with the radius of 2 cm. Subsequently, the XRD patterns of various films were determined by an X-ray diffractometer (XRD-6100, Shimadzu Corporation, Japan) using Cu-K radiation source. The voltage, electricity, scanning angular range and speed were 40 kV, 30 mA, 5–40° (2*θ*) and 4°/min, respectively.

### 2.6. Thermal stabilities of various films

The thermal stabilities of various films were determined by a NETZSCH DSC 214 (Netzsch Co, Selb/Bavaria, Germany) under a nitrogen (N_2_) atmosphere with a flow rate of 50 mL/min according to the previous methods described in the literatures [25,30] with minor adjustments. Prior to use, various films were trimmed to small fragments. Subsequently, these fragments (≈10 mg) were sealed in the sample cell. Finally, DSC curves of various films were acquired in a single heating cycle by heating from 35 to 200°C with a rate of 10°C/min.

### 2.7. Microstructure of various films

A SU-8010 SEM (Hitachi Ltd., Tokyo, Japan) was employed to study the surface morphologies of various films at an accelerating voltage of 10 kV. Before the analysis, each film was cropped to small fragments with the size of 2 cm × 2 cm and then was coated with gold under vacuum. SEM images of various films were acquired at 1000-fold magnification.

Prior to analysis, various films were trimmed to a square with the size of 5 mm × 5 mm, followed by fixing on the sample table. Then, the surface roughness analysis of various films was performed in tapping mode using an atomic force microscope (AFM) (Bruker Dimension ICON, Bruker, Germany) at room temperature. Finally, NanoScope software version 5.31 was employed to analyze the roughness parameters of various films such as root-mean-square roughness (Rq) and average roughness (Ra).

### 2.8. Mechanical properties of various films

Prior to analysis, various films were trimmed to rectangular strips with the size of 20 mm × 70 mm and the thicknesses of these trips were measured by a spiral micrometer (DL9325, Zhejiang Deli Tools Co., Ltd, Zhejiang, China). Subsequently, a CT3−4500 Texture Analyzer (Ametek Corporation, United States) was used to determine the mechanical properties of these trips from the corresponding films at a testing speed of 0.5 mm/s with a trigger point load of 0.1 N, a strain rate of 0.01 s^-1^ and an initial grip length of 50 mm. The tensile strength (TS, MPa) and elongation at break (EB, %) values of various films were calculated using the following equations:


$$TS=\frac{F}{S}$$ (1)

$$EB=\frac{L-L_{0}}{L_{0}}\times 100\%$$ (2)

where F (N), S (mm^2^), L (mm) and L_0_ (mm) are the maximum force, the cross-sectional area, the final and initial lengths of various films, respectively.

### 2.9. WVP values of various films

The WVP values of various films were determined according to the previous literatures [31,32] with some modifications. The glass cups were loaded with anhydrous calcium chloride (3 g), covered with various films, and put into a constant temperature and humidity box with an air velocity in chamber of 0.5 m·s^-1^, a relative humidity of 90% and a temperature of 25 ± 0.5°C. During the tests, the glass cups were weighed every hour interval until the mass change kept steady (less than 5%). The WVP values (g ∙ m^-1^·s^-1^·Pa^-1^) were obtained by the following equation:


$$WVP=\frac{\Delta m\times d}{S\times \Delta t\times \Delta P}$$ (3)

where d (m), S (m^2^), ΔP (Pa) and Δm/Δt (g ∙ s^-1^) are the thickness and effective area of various films, the difference in water vapor pressure across various films and the weight variation of the glass cups versus time, respectively.

### 2.10. Water solubility (WS) of various films

Firstly, various films (2 cm × 2 cm) were dried at 105°C for 24 h and weighed. Then, various dried films were placed in glass Petri dishes containing 30 mL of distilled water at 25°C for 24 h. Finally, various residue films were dried in an oven at 105°C until reaching a constant weight. The water solubility (WS) was calculated by using Eq. 4:


$$WS\left(\%\right)=\frac{W_{1}-W_{2}}{W_{1}}\times 100$$ (4)

where W_1_ (g) means the weight of various films before the immersion, W_2_ (g) means the weight of various films after immersing and drying.

### 2.11. Water contact angle (WCA) of various films

A fully automatic contact angle measuring instrument (SL250, KINO Scientifc Instrument Inc., Boston, MA, USA) was employed to determine the WCA of various films at room temperature according to the sessile drop method. Prior to analysis, various films were trimmed to a rectangle with the size of 10 mm × 20 mm, followed by fixing on the sample table. Subsequently, the surfaces of various films were lightly dropped with approximately 10 μL of distilled water using a high-precision injector. Finally, the photos of the droplets were captured after stabilizing for 30 s and their contour data were fitted using the Young-Laplace equation.

### 2.12. The potential formation mechanism of KS-FA films by molecular docking analysis

Molecular docking was employed to analyze the intermolecular interactions between SPI and KGM or FA for elucidating the potential formation mechanism of KS-FA films. SPI is mainly composed of 7S and 11S globulins. The crystal structures of 7S globulin (PDB ID: 3AUP) and 11S globulin (PDB ID: 1FXZ) were achieved from the RSCB Protein Data Bank. The protein was pretreated using PYMOL (version 2.3.4) software for the removal of water and monomer. After that, AutoDock tools (version 1.5.7) were utilized to add hydrogens, balance charge and so on for obtaining receptor molecules. Additionally, the molecular structures of KGM, LA and SA as the ligand were acquired using ChemDraw 20.0 software. Subsequently, molecular docking analysis was performed using AutoDock Vina (version 1.1.2) software and the best binding site was chosen according to binding energy. Finally, YMOL (version 2.3.4) and Ligplot (version 1.4) softwares were used to visualize the docking results.

### 2.13. Statistical analysis

All the trials were performed three times and the experimental data were reported as the means ± standard deviations (SD). Origin 8.0 software and SPSS 26 software were employed to analyze the statistical data by Least significant differences (LSD) multiple comparison tests, Duncan’s multiple range tests, and the analysis of variance.

## 3. Results and discussion

### 3.1. Rheological properties of various film solutions

**Fig 1:**
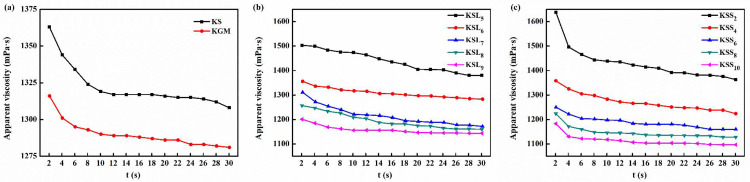
Rheological properties of various film solutions. **(a)** KGM and KS film solutions; **(b)** KSL film solutions; **(c)** KSS film solutions.

The rheological properties of KGM, KS and KS film solutions with different types and concentrations of fatty acids are displayed in Fig 1. As shown in Fig 1, all the film solutions showed the typical pseudoplastic behavior as their viscosities decreased gradually with increasing shear time. This may be probably due to the decomposition and rearrangement of molecular chains occurring in the film solutions under shear force [33,34]. It was also observed that the viscosities of KSL_5_ and KSS_2_ film solutions were higher than that of KS film solution. However, KSL and KSS film solutions exhibited lower viscosities than KS film solution when LA and SA concentrations exceeded 0.5% and 0.02%, respectively. Moreover, it was found that the viscosities of KSL and KSS film solutions decreased as FA concentrations increased. This was probably because solvent quality was reduced and intermolecular interactions between KGM and SPI were destroyed after adding fatty acids. There was no much difference in the viscosity of KS film solution between the test fatty acids. Therefore, it could be concluded that the viscosity of KS film solution was not affected by the types of fatty acids, but could be improved after incorporating appropriate concentrations of fatty acids.

### 3.2. FTIR analysis of various films

**Fig 2:**
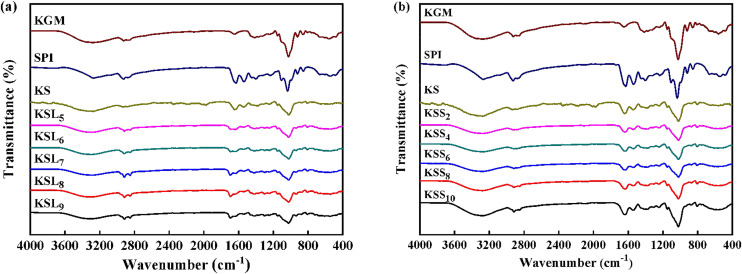
FTIR spectra of various films. **(a)** KGM, SPI, KS, KSL films; **(b)** KGM, SPI, KS, KSS films.

Fig 2 shows the infrared spectra of KGM, SPI, KS and KS films with different types and concentrations of fatty acids. The spectrum of pure KGM film exhibited characteristic absorption peaks at 3282, 2921, 2853, 1646 and 1025 cm^-1^, which were attributed to O-H stretching, -CH_2_ bond stretching, -CH bond stretching, intramolecular hydrogen bonds and C-O stretching, respectively. In addition, the absorption bands at 854 and 807 cm^-1^ were associated with mannose groups stretching vibration in KGM [3,35]. In pure SPI film spectrum, the absorption peaks at 1628, 1540, and 1236 cm^-1^ were ascribed to C = O stretching vibration in the amide I, N-H bending vibration in the amide II, C-N and N-H stretching vibration in the amide III, respectively [11,36]. Moreover, the characteristic absorption peaks of pure SPI film at 3271, 2926, 2877, 1402 and 1035 cm^-1^ were attributed to the vibrations of O-H stretching, -CH_2_ bond stretching, -CH bond stretching, CH_2_ bending and C-O stretching, respectively [37].

It was found that the characteristic absorption peaks of pure KGM and SPI films all appeared in the spectra of KS, KSL and KSS films. Nevertheless, compared to KS film, the absorption peak of O-H stretching vibrations shifted gradually to higher wavenumber after adding fatty acids, indicating that hydrogen bonds were formed among KGM, SPI, and fatty acids. Moreover, a higher FA concentration made the characteristic absorption peaks at around 2917 and 2847 cm^-1^ in the spectra of KS-FA films more apparent, suggesting the presence of hydrophobic interactions. It was also observed that the intensity of C = O stretching vibration peak in the spectra of KS-FA films increased with the increase of FA concentrations. However, the C = O absorption peak position shifted to higher wavenumber in the spectra of KSL films. This may be probably because the agglomerations of LA with high additive amounts floated on the film surface during the preparation progress, which was consistent with the stretching vibration of the carbonyl groups of LA [29,38]. In addition, the intensity of C-O stretching vibration peak in the spectra of KSS films increased with the increase of SA concentrations whereas it kept constant in the spectra of KSL films. This may be probably because KSL films had a higher ordered degree of short-range structure [24]. The above results revealed that the chemical structures of KGM, SPI and fatty acids in the ternary composite films had no notable changes, but the mechanical and water vapor barrier properties of KS-FA films could be affected by the intermolecular interactions among the three components.

### 3.3. XRD analysis of various films

**Fig 3:**
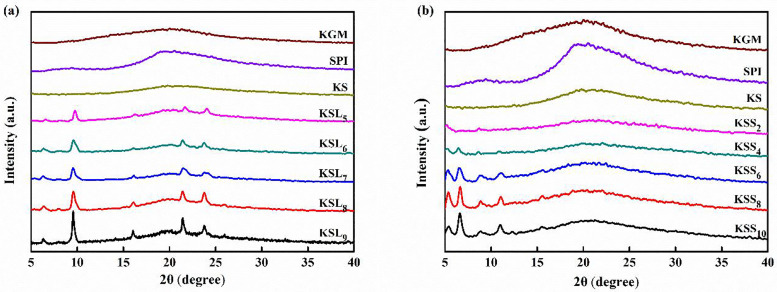
XRD patterns of various films. **(a)** KGM, SPI, KS, KSL films; **(b)** KGM, SPI, KS, KSS films.

The XRD patterns of KGM, SPI, KS and KS films with different types and concentrations of fatty acids are shown in Fig 3. A broad diffraction peak with amorphous structure characteristic at 2θ = 20° was observed in the XRD pattern of pure KGM film [30,39]. Pure SPI film exhibited two broad and smooth diffraction peaks at 2θ = 9.3° and 20.4°. This result was similar to the previous reports [16,40]. After adding SPI to pure KGM film, the diffraction peak at 2θ = 9.3° disappeared and the peak at 2θ = 20.5° became more flat, which may due to the good compatibility of KGM and SPI in the composite film.

After further adding LA to KS film, the diffraction peak at 2θ = 20.5° disappeared. In addition, five new characteristic diffraction peaks at 2θ = 6.4°, 9.6°, 16.1°, 21.4° and 23.8° were observed in the XRD patterns of KSL films and their intensities increased with the increase of LA concentrations. Meanwhile, it was found that the peak intensity of KSS films at 2θ = 20.5° slightly decreased when the concentrations of SA were 0.02 and 0.04%, respectively. However, KSS films had similar peak intensities to KS film at 2θ = 20.5° when the concentrations of SA exceeded 0.04%. Furthermore, KSS films also displayed five new diffraction peaks at 2θ = 5.4°, 6.6°, 8.8°, 11°, 15.5°, and the intensities of these peaks increased with the increase of SA concentrations. More importantly, there was no significant change in the crystalline peak position of KS films with different concentrations of fatty acids. Therefore, it could be concluded that the crystalline structure of KS film was improved after incorporating fatty acids.

### 3.4. DSC analysis of various films

**Fig 4:**
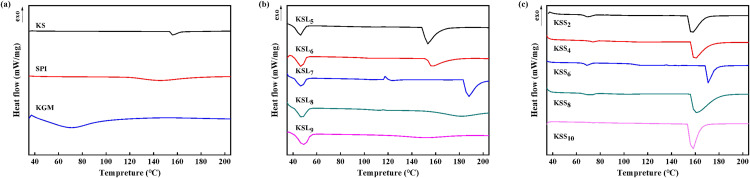
DSC curves of various films. **(a)** KGM, SPI, KS films; **(b)** KSL films; **(c)** KSS films.

Fig 4 shows the DSC curves of KGM, SPI, KS and KS films with different types and concentrations of fatty acids. A single endothermic peak at around 71, 146 and 155°C was observed in the DSC curves of KGM, SPI and KS films, respectively, which corresponded to the respective glass transition temperature (Tg). This result suggested that the thermal stability of KS film was higher than those of pure KGM and SPI films, which may be due to the formation of a dense network structure by the intermolecular interactions between KGM and SPI.

As for the DSC curves of KSS and KSL films, the endothermic peaks at about 68 ~ 72°C and 47°C were attributed to the melting points of SA and LA, respectively [25,41] Moreover, it was found that all the KSS and KSL films exhibited a single Tg at around 158 ~ 172 and 150 ~ 190°C, respectively, which suggested good compatibility among KGM, SPI and fatty acids. However, the Tg of KS-FA films increased first and then decreased with the increase of FA concentrations. This may be probably because the carboxyl groups of FAs with small quantities preferentially formed hydrogen bonding networks with the polar groups (-OH, -NH_2_) of KGM and SPI molecules, but the uneven distribution of excessive FAs weakened the intermolecular interactions, increased the free volume and exhibited typical plasticizing effects. It was discovered that the Tg of KSL_5_ and KSL_9_ films was lower than that of KS film, whereas the other KSL films and all the KSS films had higher Tg than KS film. The Tg of KSS and KSL films reached the maximum values (170 and 188°C) when the concentrations of SA and LA were 0.06% and 0.7%, respectively. These results indicated that the thermal stability of KS film could be significantly improved after incorporating appropriate concentrations of fatty acids.

### 3.5. Microstructural analysis of various films

**Fig 5:**
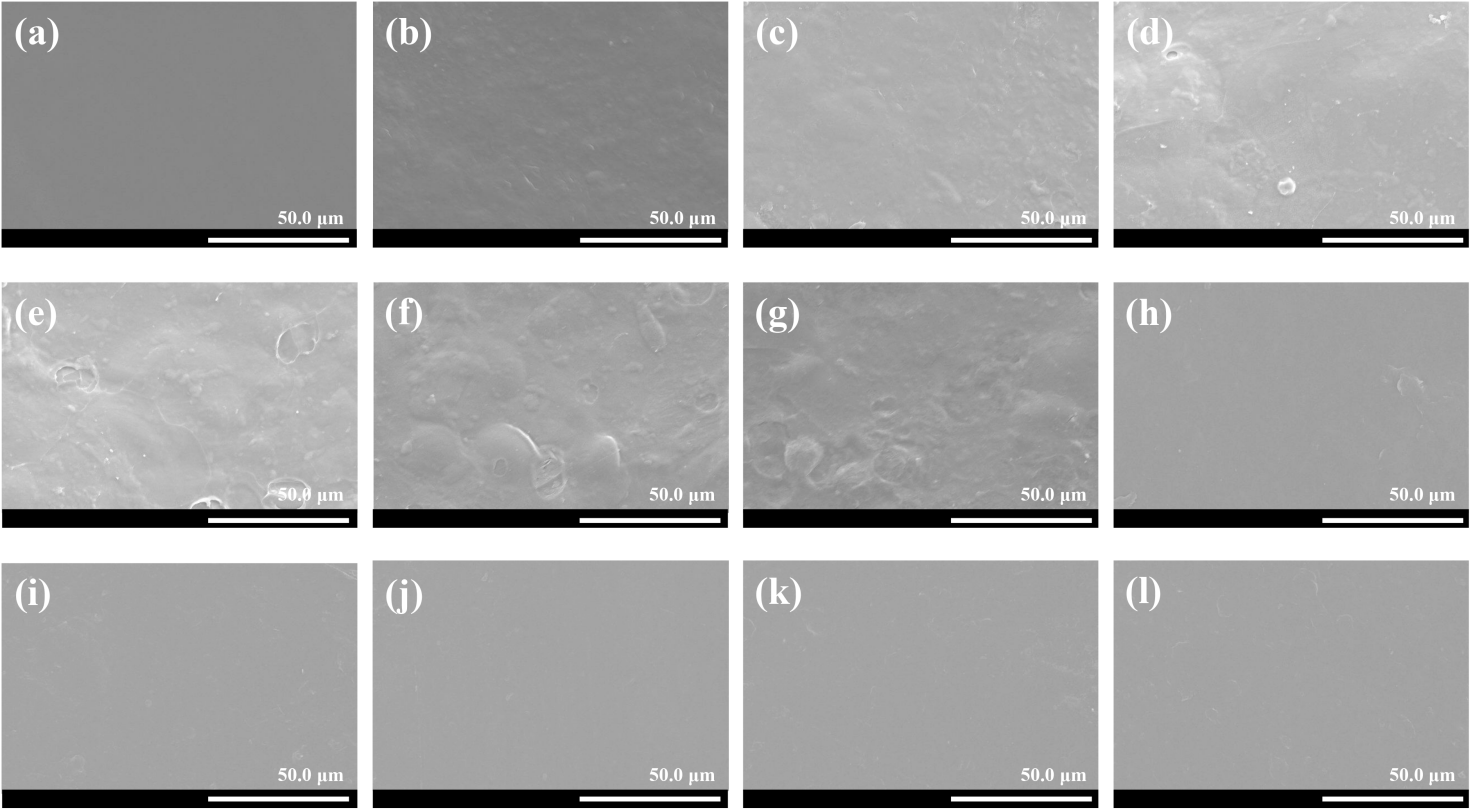
SEM micrographs of various films. **(a)** Pure KGM film; **(b)** KS film; **(c)** KSL_5_ film; **(d)** KSL_6_ film; **(e)** KSL_7_ film; **(f)** KSL_8_ film; **(g)** KSL_9_ film; **(h)** KSS_2_ film; **(i)** KSS_4_ film; **(j)** KSS_6_ film; **(k)** KSS_8_ film; **(l)** KSS_10_ film.

The SEM micrographs of KGM, KS and KS films with different types and concentrations of fatty acids are presented in Fig 5. It was observed that pure KGM film had a smooth and homogeneous surface, indicating the good compatibility of KGM and glycerol. A similar phenomenon was observed in the previous reports [42,43]. However, KS film displayed a little rough surface, which may be due to the agglomerations of the polymers and the intermolecular interactions between KGM and SPI in the film matrix [44].

As for KSL films, the surfaces became much rougher and more irregular when compared to KS film. Moreover, the surface roughness and irregularity of KSL films increased as LA concentrations increased. When LA concentration was 0.9%, the surface of KSL film was the roughest and most irregular. This may be probably because the agglomerations of LA were unevenly embedded into the KS film matrix and may float on the surface during the preparation progress [29]. Although KSS films showed relatively a little rougher surfaces than KS film, their surface roughness and irregularity presented an irregular trend (increased first, then decreased, and again increased) with the increase of SA concentrations. When SA concentration was 0.06%, the surface roughness and irregularity of KSS film were the least noticeable. This may be probably because of the good compatibility and strong intermolecular interactions between SA and KS film matrix at this SA concentration. These results showed that there were significant changes in the surface morphology of KS film after the incorporation of different fatty acids with different concentrations.

**Fig 6:**
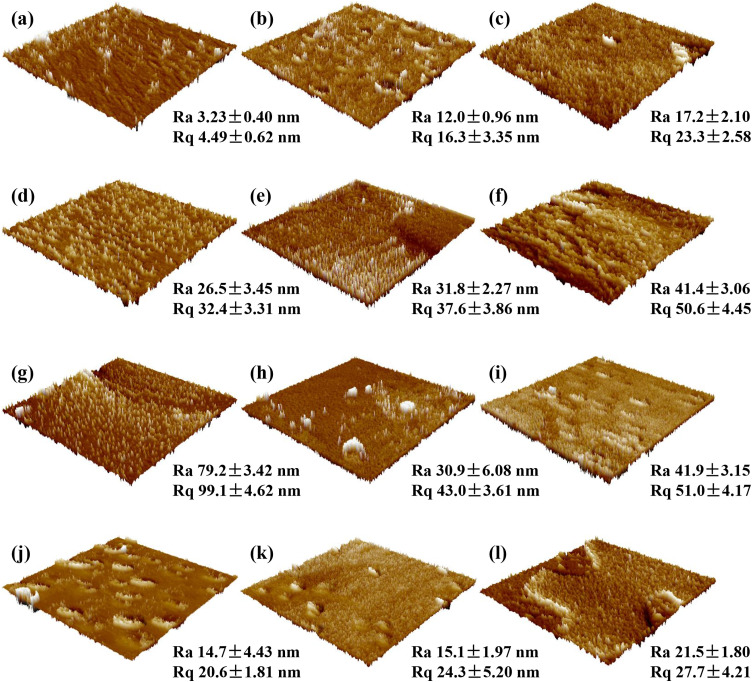
AFM images of various films. **(a)** Pure KGM film; **(b)** KS film; **(c)** KSL_5_ film; **(d)** KSL_6_ film; **(e)** KSL_7_ film; **(f)** KSL_8_ film; **(g)** KSL_9_ film; **(h)** KSS_2_ film; **(i)** KSS_4_ film; **(j)** KSS_6_ film; **(k)** KSS_8_ film; **(l)** KSS_10_ film.

The AFM images of KGM, KS and KS films with different types and concentrations of fatty acids are showed in Fig 6. It was found that pure KGM film possessed low Ra and Rq values, suggesting that its surface was relatively homogenous and smooth. However, the surface roughness of pure KGM film was enhanced after incorporating SPI. In addition, it was observed that the Ra and Rq values of KS-FA films were higher than those of KS film, indicating that KS-FA films exhibited higher surface roughness than KS film. Nevertheless, the Ra and Rq values of KS films with different fatty acids had different variation patterns. The Ra and Rq values of KSL films increased as LA concentrations increased, whereas the Ra and Rq values of KSS films showed an irregular trend (increased first, then decreased, and again increased) with the increase of SA concentrations. KSL film had the highest Ra and Rq values when LA concentration was 0.9%. KSS_6_ film had the lowest Ra and Rq values when compared with other KS-FA films. These results showed that adding different fatty acids with different concentrations significantly affected the surface roughness of KS film, as consistent with SEM results.

### 3.6. Mechanical properties of various films

The mechanical properties of KGM, SPI, KS, KS films with different types and concentrations of fatty acids are shown in Table 1. It was observed that the TS and EB values of KS film were 25.33 ± 0.51 MPa and 7.61 ± 0.91%, respectively, which were significantly increased (p < 0.05) when compared to pure KGM film. This may be probably because a dense network structure was formed as a consequence of the intermolecular interactions between KGM and SPI. In addition, it was found that the TS values of KS-FA films increased first and then decreased with the increase of FA concentrations, while their EB values showed an irregular trend (decreased first, then increased and again decreased). The TS and EB values of KSS and KSL films reached the maximum values when the concentrations of SA and LA were 0.06% and 0.7%, respectively. More importantly, it was discovered that KSL_7_ film displayed higher TS and EB values (27.75 MPa and 11.59%) than other KGM-based films. There were a 1.08-fold increase of TS value and a 1.52-fold increase of EB value in KSL_7_ film when compared with KS film. These results showed that the TS (F_0.05_(12,26)=228.15, p = 3.70626 × 10^-23^ < 0.001) and EB values (F_0.05_(12,26)=611.94, p = 1.10621 × 10^-28^ < 0.001) of KS film were affected by the concentrations and types of fatty acids.

**Table 1:** Mechanical properties of KGM, SPI, KS, KS films with different types and concentrations of fatty acids.

Films	TS/MPa	EB/%
KGM	21.83 ± 0.05^cd^	3.03 ± 0.13^f^
SPI	3.59 ± 0.23^g^	32.34 ± 0.36^a^
KS	25.33 ± 0.51^b^	7.61 ± 0.91^c^
KSL_5_	15.49 ± 0.43^f^	5.29 ± 0.10^d^
KSL_6_	20.12 ± 0.17^e^	4.03 ± 0.63^e^
KSL_7_	27.75 ± 0.59^a^	11.59 ± 0.23^b^
KSL_8_	22.43 ± 0.24^c^	7.69 ± 0.16^c^
KSL_9_	20.65 ± 1.20^de^	3.09 ± 0.04^ef^
KSS_2_	21.97 ± 0.97^c^	7.46 ± 0.87^c^
KSS_4_	24.63 ± 0.60^b^	5.27 ± 0.10^d^
KSS_6_	27.45 ± 0.36^a^	7.54 ± 0.69^c^
KSS_8_	22.82 ± 0.68^c^	3.81 ± 0.27^ef^
KSS_10_	22.55 ± 0.46^c^	3.78 ± 0.79^ef^

### 3.7. Water solubility (WS) and vapor permeability (WVP) of various films

The WVP and WS results of KGM, SPI, KS, KS films with different types and concentrations of fatty acids are displayed in Table 2. As seen from Table 2, the WVP value of KS film was higher than that of pure KGM film, but was significantly lower (p < 0.05) than that of pure SPI film. This may be attributed to the compact structure formed by the intermolecular interactions between KGM and SPI. In addition, it was observed that the WVP values of KS-FA films decreased first and then increased as the concentrations of FA increased. The WVP values of KSS and KSL films reached the minimum values when the concentrations of SA and LA were 0.06% and 0.7%, respectively. Compared to KS film, the water vapor barrier properties of KSS_6_ and KSL_7_ films had been improved by 48.6% and 43%, respectively. Moreover, it was discovered that KSL_7_, KSL_8_ and all the KSS films had lower WVP values than KS film, which meant that these KS-FA films had better water vapor barrier properties than KS film. It could be concluded that the water vapor barrier properties of KS film were affected by the types of fatty acids and could be enhanced after incorporating appropriate concentrations of fatty acids (F_0.05_(12,26)=97.91, p = 1.77663 × 10^-18^ < 0.001).

**Table 2:** The WVP and WS values of KGM, SPI, KS, KS films with different types and concentrations of fatty acids.

Films	WVP × 10^−10^ (g ∙ m^-1^·s^-1^·Pa^-1^)	WS (%)
KGM	0.99 ± 0.00^b^	87.40 ± 0.39^a^
SPI	2.02 ± 0.14^a^	25.05 ± 0.02^g^
KS	1.07 ± 0.01^b^	31.57 ± 0.20^b^
KSL_5_	1.04 ± 0.06^b^	30.23 ± 0.64^cd^
KSL_6_	1.01 ± 0.03^b^	30.03 ± 0.33^cd^
KSL_7_	0.61 ± 0.03^ef^	27.00 ± 0.06^f^
KSL_8_	0.73 ± 0.12^d^	28.80 ± 0.25^e^
KSL_9_	1.05 ± 0.01^b^	30.31 ± 0.33^c^
KSS_2_	0.78 ± 0.05^cd^	28.91 ± 0.08^e^
KSS_4_	0.69 ± 0.06^de^	27.13 ± 0.05^f^
KSS_6_	0.55 ± 0.10^f^	26.75 ± 0.75^f^
KSS_8_	0.76 ± 0.04^cd^	28.86 ± 0.32^e^
KSS_10_	0.86 ± 0.00^c^	29.68 ± 0.29^d^

It was also observed from Table 2 that the WS value of pure KGM film reached 86.46%, suggesting that pure KGM film exhibited strong hydrophilicity. This may be probably because the chain of KGM molecules had a large number of hydroxyl groups. Incorporating SPI significantly (p < 0.05) decreased the WS value of pure KGM film, suggesting that KS film had better water resistance properties than pure KGM film. In addition, it was discovered that the WS values of KS-FA films decreased first and then increased with the increase of FA concentrations, but were significantly lower (p < 0.05) than that of KS film, which meant that KS-FA films had better water resistance properties than KS film. KSL and KSS films had the lowest WS values when LA and SA concentrations were 0.7% and 0.06%, respectively. Compared to KS film, the water resistance properties of KSS_6_ and KSL_7_ films had been improved by 15.3% and 14.5%, respectively. However, there was no much change in the WS value of KS film among different types of fatty acids. More importantly, it was found that compared to other KGM-based films, KSS_6_ film had the best water resistance properties. These results indicated that the water resistance properties of KS film could be improved after adding fatty acids with appropriate concentrations (F_0.05_(12,26)=6347.16, p = 7.27823 × 10^-42^ < 0.001).

### 3.7. Water contact angle (WCA) of various films

**Fig 7:**
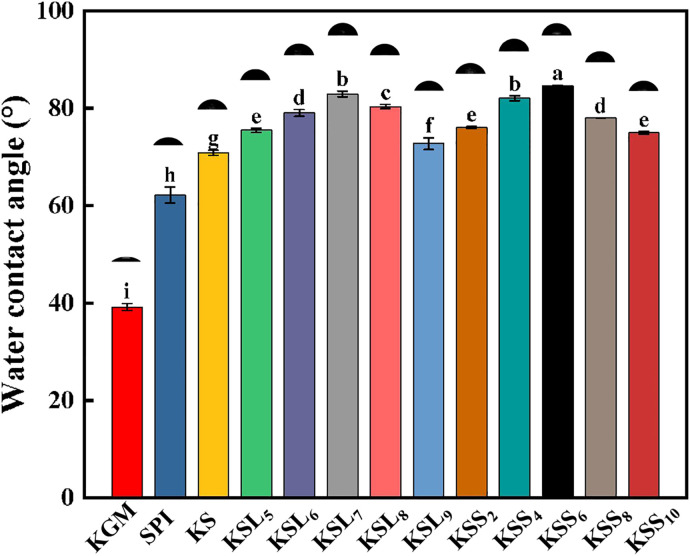
WCA results of various films. Different lower-case letters indicate significant differences after Duncan’s multiple comparison test (p ≤ 0.05), while the same letter indicates no significant differences.

Fig 7 shows the WCA results of KGM, SPI, KS, KS films with different types and concentrations of fatty acids. It was observed that pure KGM film had low WCA value (39.21°). However, the WCA value of pure KGM film was significantly (p < 0.05) increased after incorporating SPI. This may be due to the formation of intermolecular interactions between KGM and SPI molecules. In addition, it was found that the WCA values of KS-FA films increased first and then decreased with the increase of FA concentrations, but were significantly higher (p < 0.05) than that of KS film. KSL and KSS films had the highest WCA values when LA and SA concentrations were 0.7% and 0.06%, respectively. Compared to KS film, the WCA values of KSS_6_ and KSL_7_ films had been improved by 19.4% and 17%, respectively. However, there was no much change in the WCA value of KS film among different types of fatty acids. More importantly, it was discovered that compared to other KGM-based films, KSS_6_ film had the highest WCA value. This may be probably because of the strongest intermolecular interactions among KGM, SPI, and SA at this unique blending ratio. These results indicated that incorporating fatty acids could significantly increase the static water contact angle of KS film.

### 3.8. The potential formation mechanism of KS-FA films

**Fig 8:**
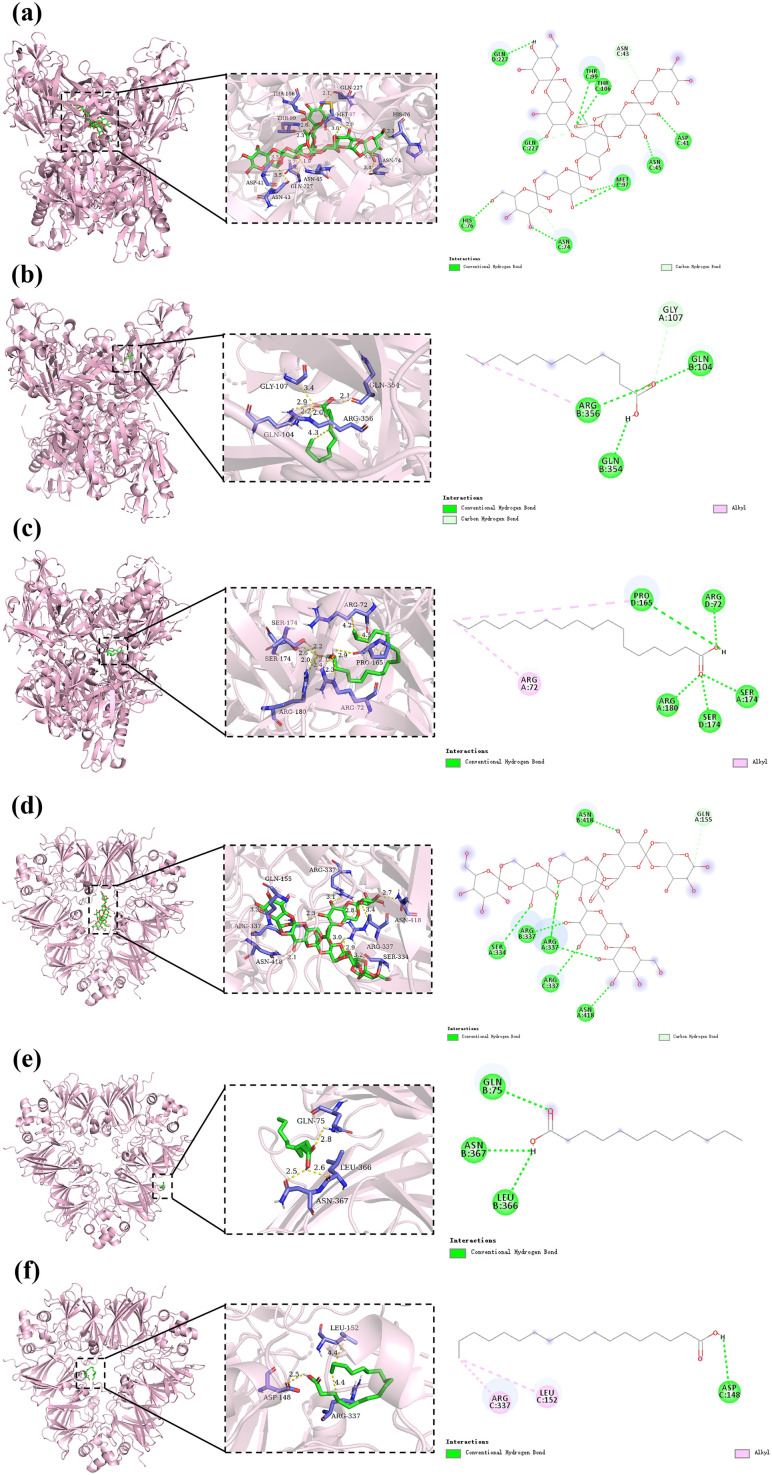
The potential formation mechanism of KS-FA films. **(a)** Interactions between 7S and KGM; **(b)** Interactions between 7S and LA; **(c)** Interactions between 7S and SA; **(d)** Interactions between 11S and KGM; **(e)** Interactions between 11S and LA; **(f)** Interactions between 11S and SA.

Fig 8 shows the docked conformations of 7S and 11S globulins with KGM, LA and SA. Their binding energies were −15.8, −5.0, −6.1, −11.6, −4.7 and −4.6 Kcal/mol, respectively. This result demonstrated the effectiveness of the docking model due to their binding energies below −1.20 Kcal/mol. In addition, it was found that the binding energies of 7S and 11S globulins with KGM, 7S globulin with LA and SA were all less than or equal to −5.00 Kcal/mol, indicating that 7S and 11S globulins with KGM, 7S globulin with LA and SA were able to form stable complexes. Moreover, the binding energies between 7S globulin with KGM, LA and SA were all lower than those of 11S globulin with KGM, LA and SA, indicating that the binding between 7S globulin with KGM, LA and SA displayed greater stabilities. Thus, the binding of SPI with KGM and FA suggested possible hydrogen bond and hydrophobic contributions from the interactions between 7S globulin with KGM and FA.

KGM formed nine conventional hydrogen bonds with Gln227, Thr99, Thr106, Asp41, Asn45, Asn74, Met97 and His76 of 7S globulin, and it had a carbon hydrogen bond, namely hydrophobic interaction, with Asn43 of 7S globulin. Similarly, six conventional hydrogen bonds (Arg337, Asn418 and Ser334) and a carbon hydrogen bond (Gln155) were also observed between KGM and 11S globulin. These findings were consistent with the results reported by the previous literatures that the interactions between KGM and SPI were mainly correlated to hydrogen bonds and hydrophobic interactions [18,45]. LA formed three conventional hydrogen bonds with Gln104, Gln354, Arg356 of 7S globulin, and Gln75, Asn367, Leu366 of 11S globulin, respectively. Moreover, carbon hydrogen bond (Gly107) and alkyl hydrophobic interaction (Arg356) were also found between LA and 7S globulin. SA formed five conventional hydrogen bonds (Pro165, Arg72, Arg180, Ser174) and two alkyl hydrophobic interactions (Pro165 and Arg72) with 7S globulin. Similarly, a conventional hydrogen bonds (Asp148) and two alkyl hydrophobic interactions (Arg337 and Leu152) were also discovered between SA and 11S globulin. These findings suggested that the intermolecular interactions between SPI and KGM or FA were mainly hydrogen bonds, accompanied by a small amount of hydrophobic interactions, consistent with FTIR results. Therefore, molecular docking results further corroborated the interaction mechanism between SPI and KGM or FA at the molecular level, enabling a deeper understanding of the underlying formation mechanism of KS-FA films. Further molecular‑dynamics simulations (e.g., 10 ns explicit‑water MD) could be performed in the future to quantitatively assess the lifetimes of these hydrogen bonds and the extent of hydrophobic contacts.

## 4. Conclusions

A serial of novel KGM/SPI/fatty acid (KS-FA) films were successfully fabricated by a solution casting method in this study. FTIR results and molecular docking suggested the formation of hydrogen bonds and hydrophobic interactions in the KS-FA films. Microstructural analysis revealed that a dense network structure with rougher surface was observed in the KS-FA films. KS-FA films had higher crystallinities and water contact angles than KS film. Moreover, incorporating fatty acids with appropriate concentrations improved the rheological properties of the film-forming solutions, thermal stabilities, water resistance, water vapor barrier and mechanical properties of KS film. KSL_7_ film had the highest thermal stability and exhibited stronger mechanical properties than other KGM-based films. The WVP and WS values of KS-FA films reached the minimum when the concentrations of SA and LA were 0.06% and 0.7%, respectively. These results indicated the great potential application of KS-FA films as biodegradable food packaging materials. Thus, this paper provides a novel perspective and establishs a foundation for the development of food packaging films with better structure and properties. However, the stability of KS-FA films in the practical packaging applications of various foods is not evaluated in this paper. Therefore, further researches will focus on studying the preservation effects and mechanisms of KS-FA films on different types of food. Moreover, the structure-property relationship and exact forming mechanism of KS-FA films will also be further explored through various advanced technologies.

## Supporting information

S1 Table Composition clarity. (XLS)

S2 Table Tg data from DSC curves. (XLS)

S3 Table Thickness distribution. (XLS)

S4 Table Film effective film area, CV of WVP and effective film area. (XLS)

S1 File Minimal data set. (ZIP)
