# Supplementary figures and images for: Development of a novel konjac glucomannan/soy protein isolate/fatty acid composite film: Insights into the structure, properties and interaction mechanism
Source: PLoS One. 2026 Feb 9;21(2):e0340257. doi: 10.1371/journal.pone.0340257 (PMC12885255; doi:10.1371/journal.pone.0340257)

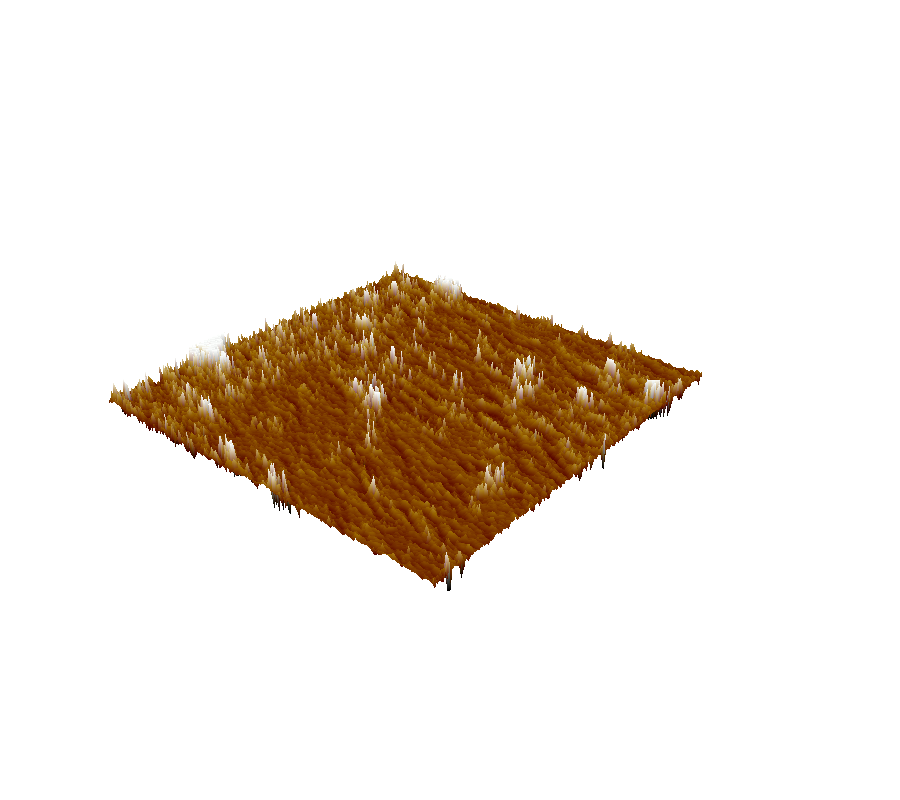

Supplement: S1 File — (ZIP) [file pone.0340257.s005.zip › S1 File/AFM images/KGM.tif]

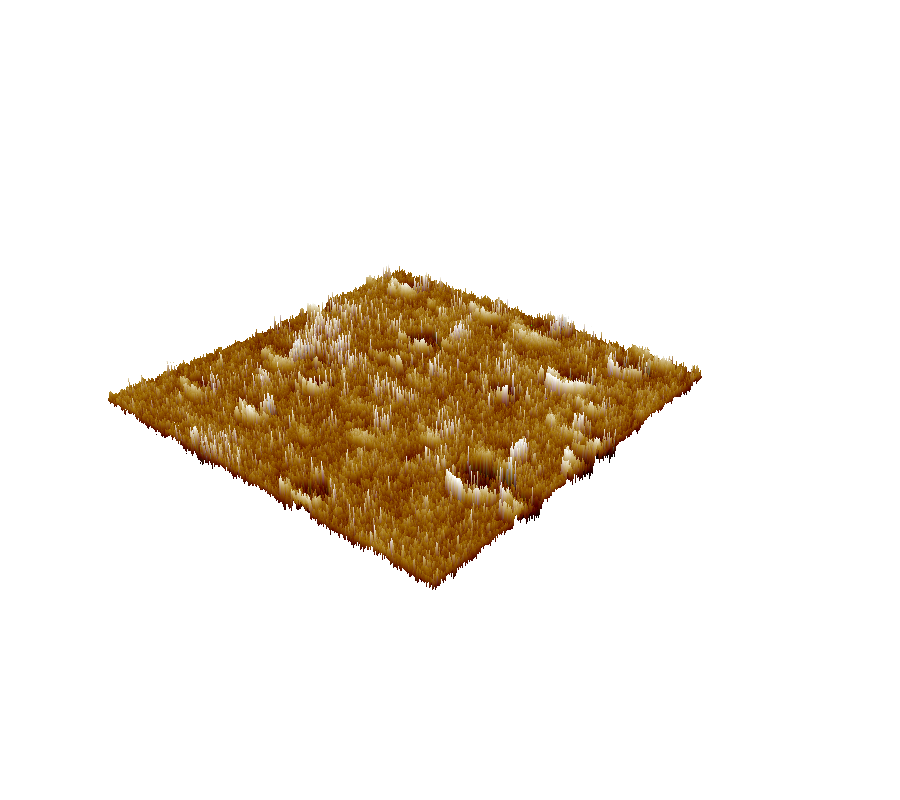

Supplement: S1 File — (ZIP) [file pone.0340257.s005.zip › S1 File/AFM images/KS.tif]

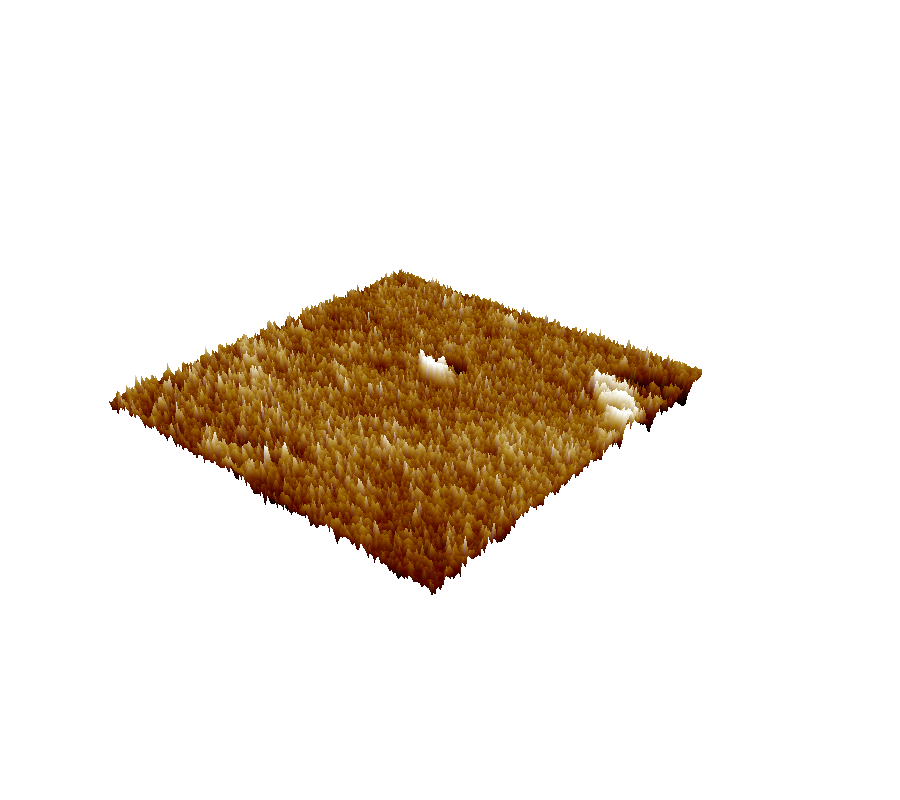

Supplement: S1 File — (ZIP) [file pone.0340257.s005.zip › S1 File/AFM images/KSL5.tif]

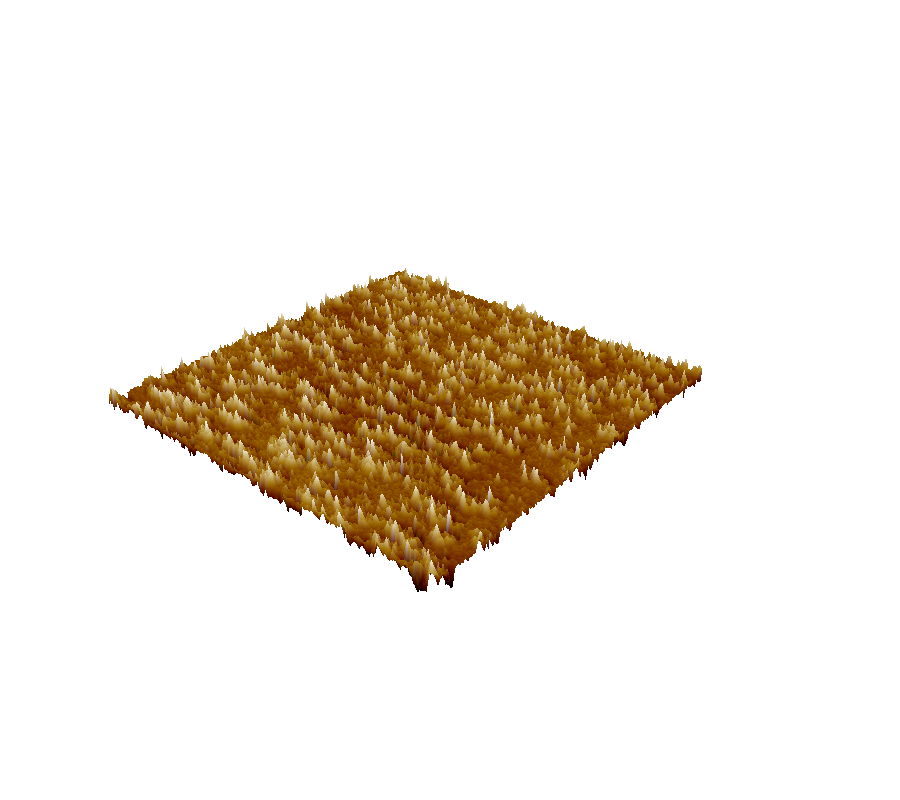

Supplement: S1 File — (ZIP) [file pone.0340257.s005.zip › S1 File/AFM images/KSL6.tif]

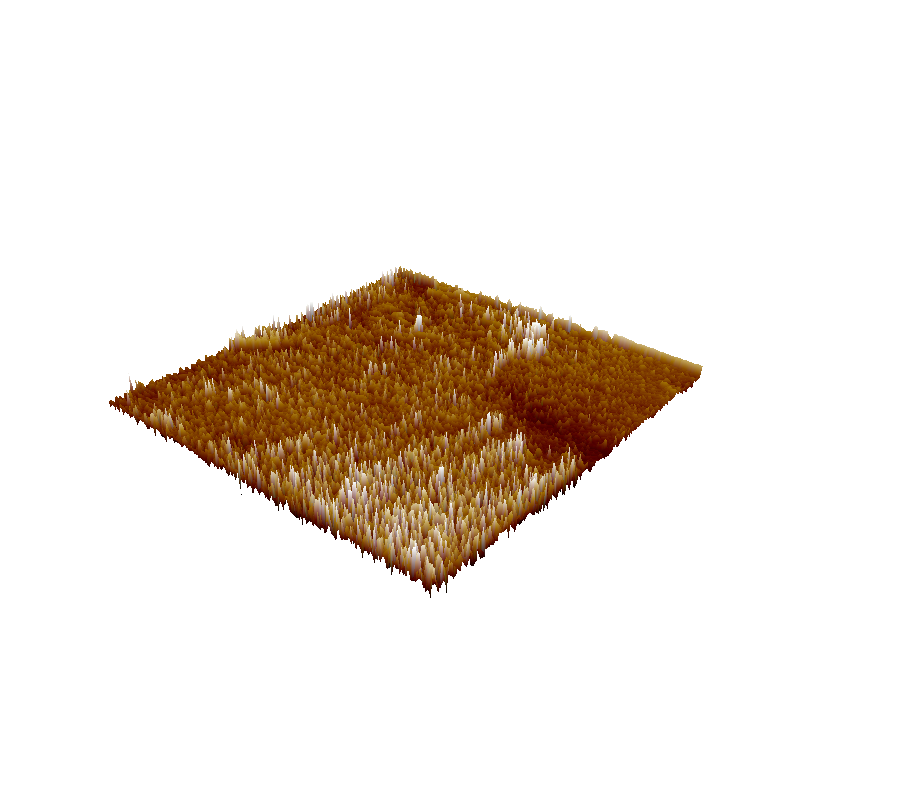

Supplement: S1 File — (ZIP) [file pone.0340257.s005.zip › S1 File/AFM images/KSL7.tif]

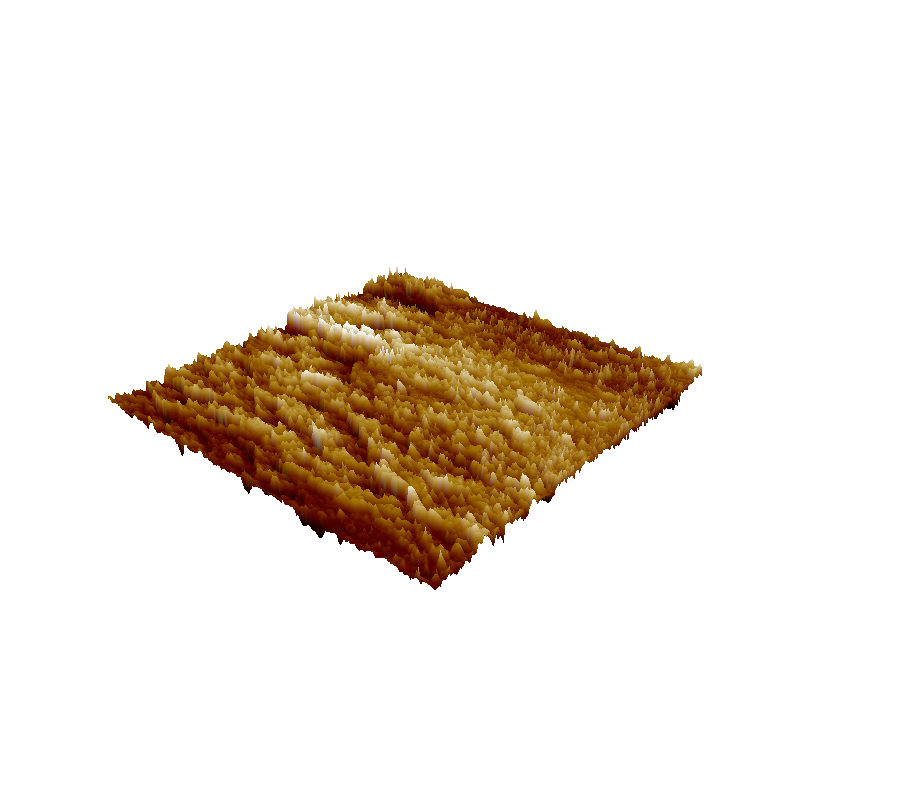

Supplement: S1 File — (ZIP) [file pone.0340257.s005.zip › S1 File/AFM images/KSL8.tif]

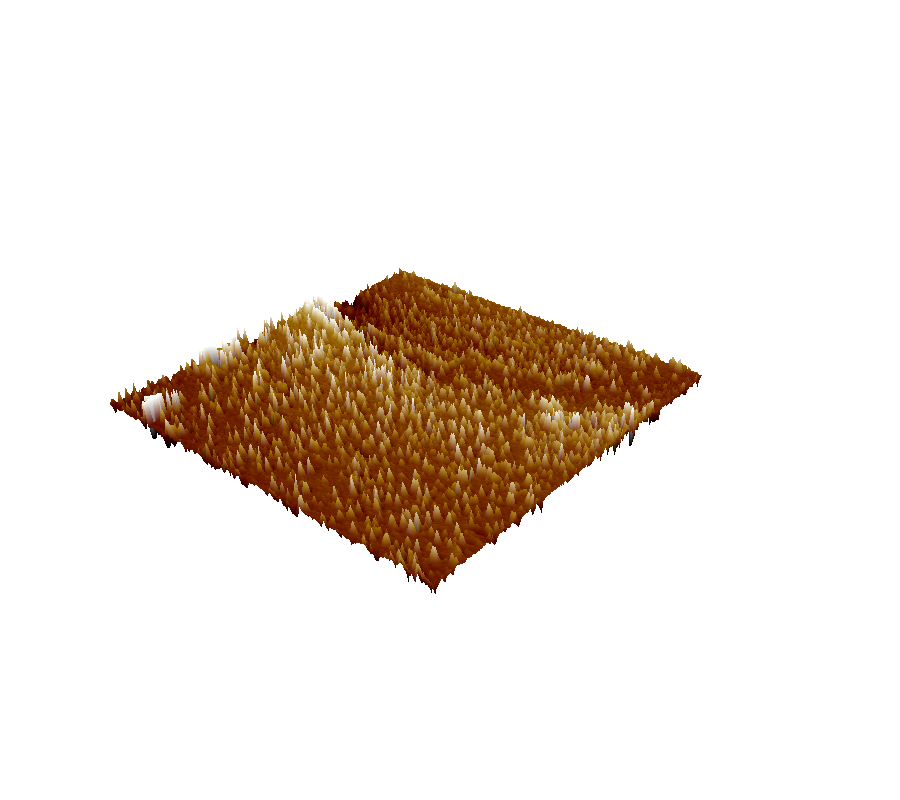

Supplement: S1 File — (ZIP) [file pone.0340257.s005.zip › S1 File/AFM images/KSL9.tif]

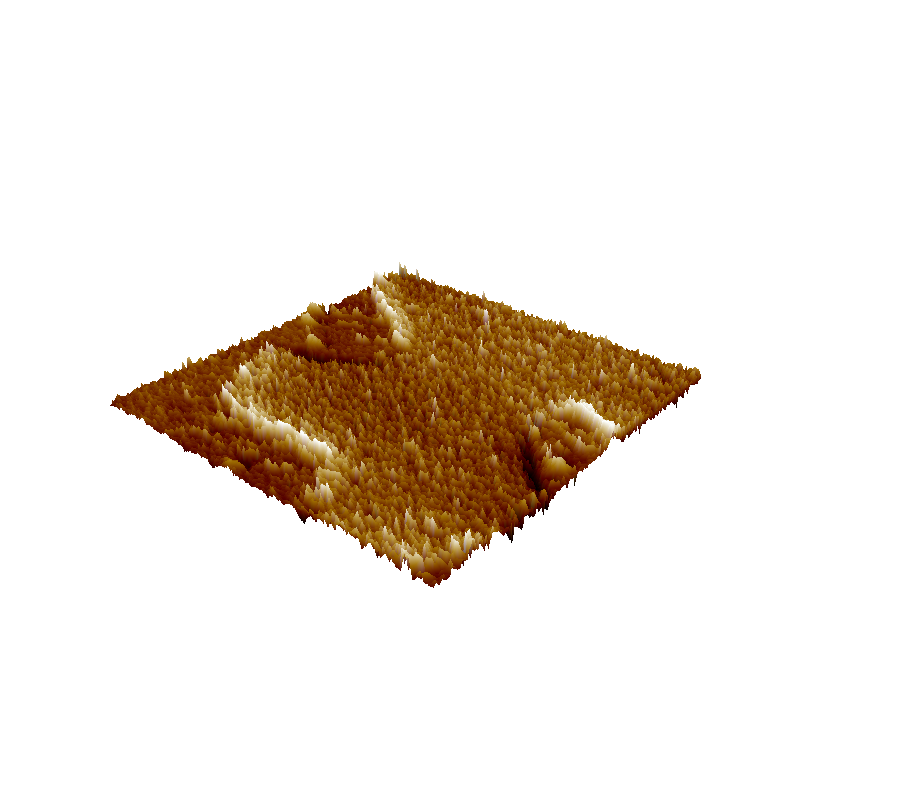

Supplement: S1 File — (ZIP) [file pone.0340257.s005.zip › S1 File/AFM images/KSS10.tif]

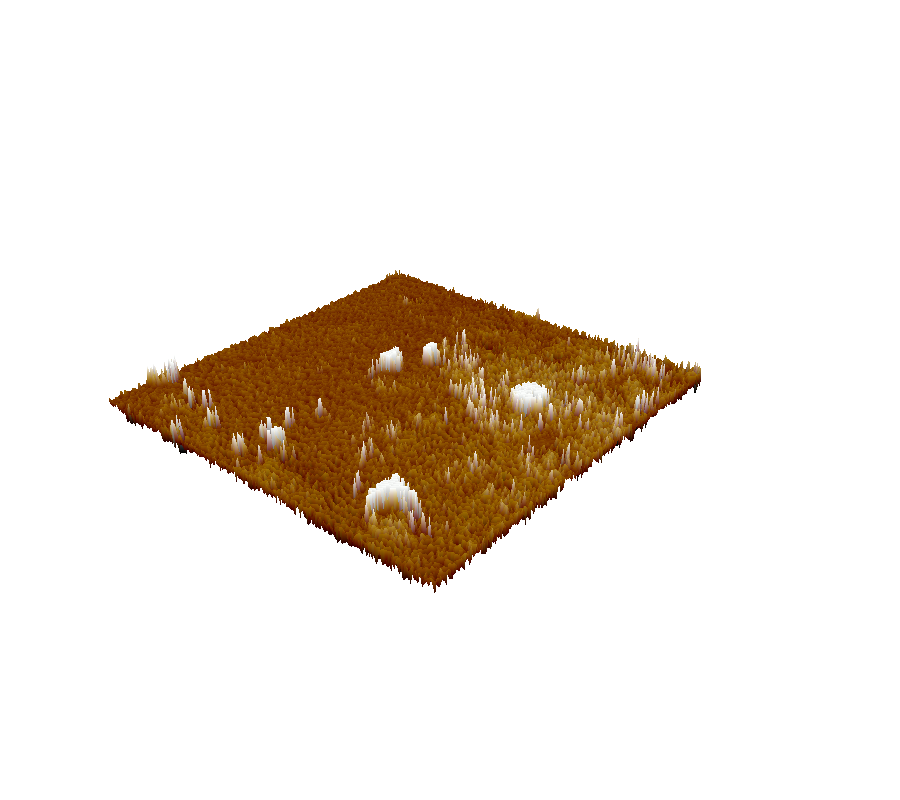

Supplement: S1 File — (ZIP) [file pone.0340257.s005.zip › S1 File/AFM images/KSS2.tif]

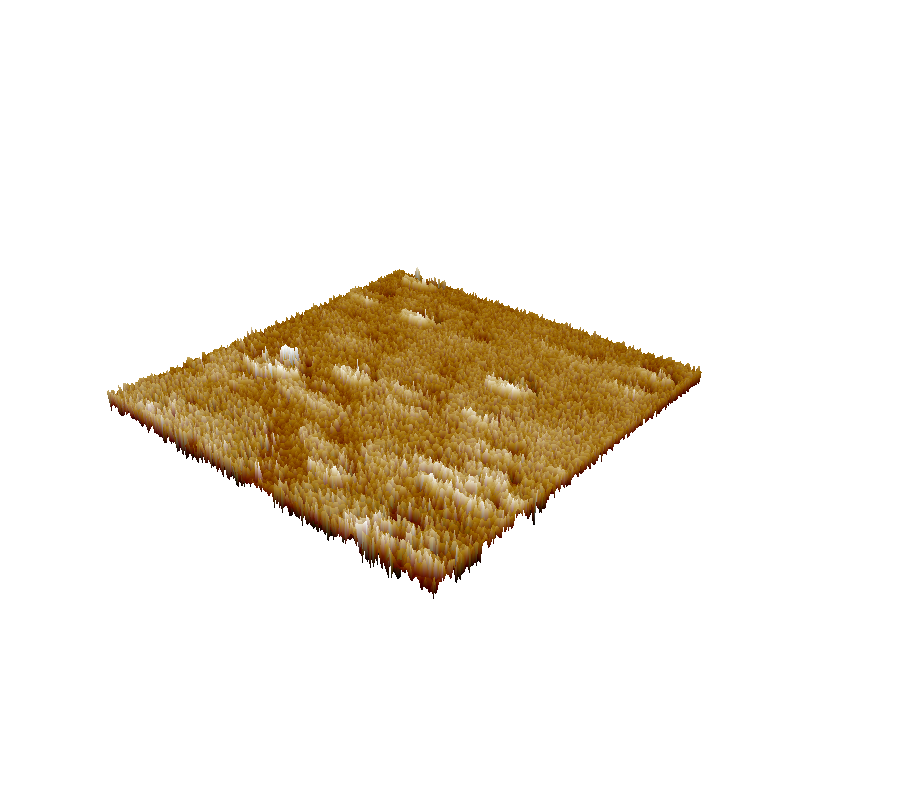

Supplement: S1 File — (ZIP) [file pone.0340257.s005.zip › S1 File/AFM images/KSS4.tif]

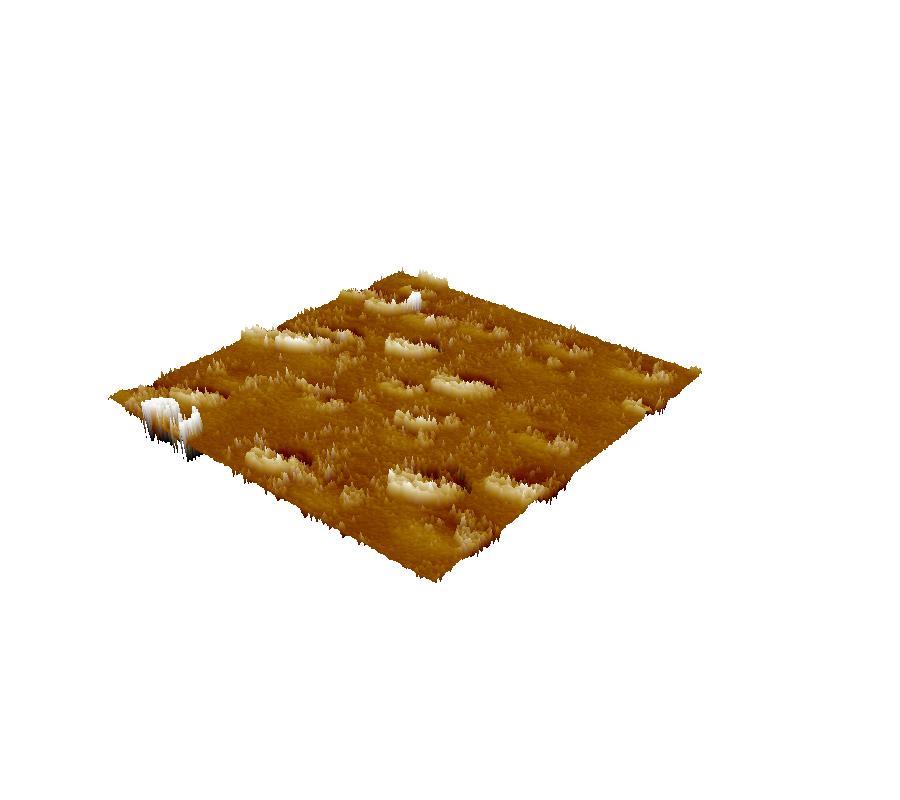

Supplement: S1 File — (ZIP) [file pone.0340257.s005.zip › S1 File/AFM images/KSS6.tif]

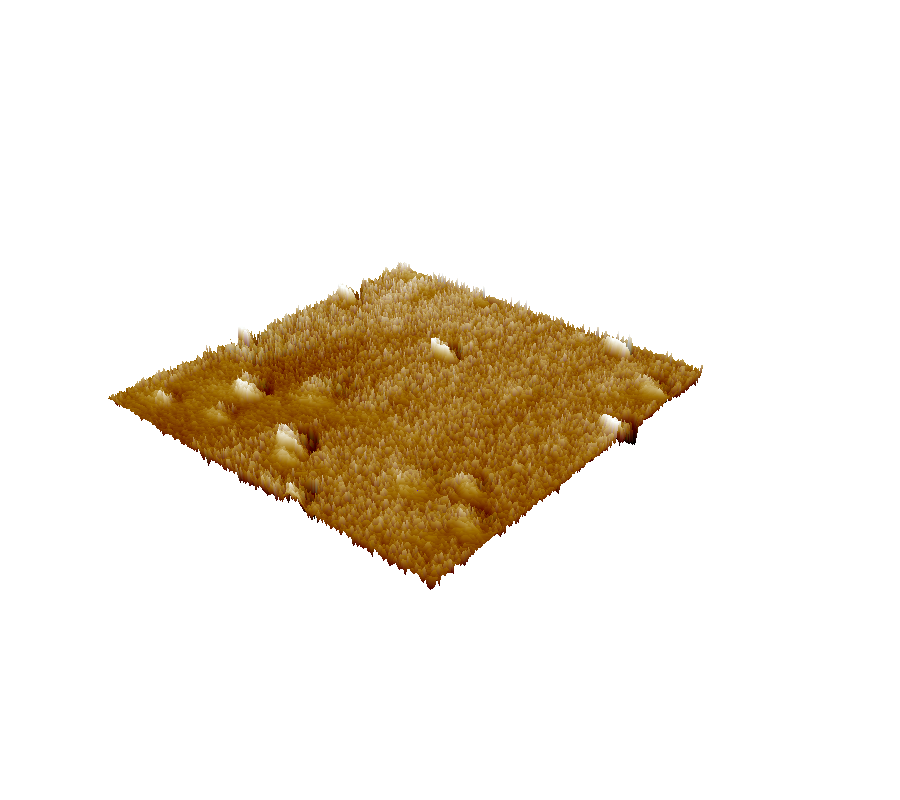

Supplement: S1 File — (ZIP) [file pone.0340257.s005.zip › S1 File/AFM images/KSS8.tif]

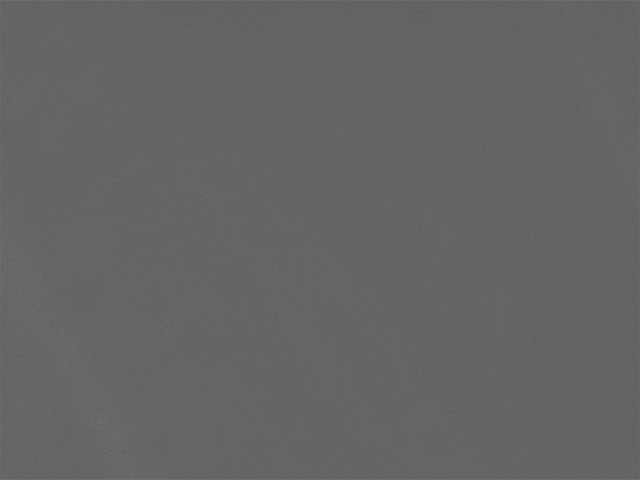

Supplement: S1 File — (ZIP) [file pone.0340257.s005.zip › S1 File/SEM images/KGM.tif]

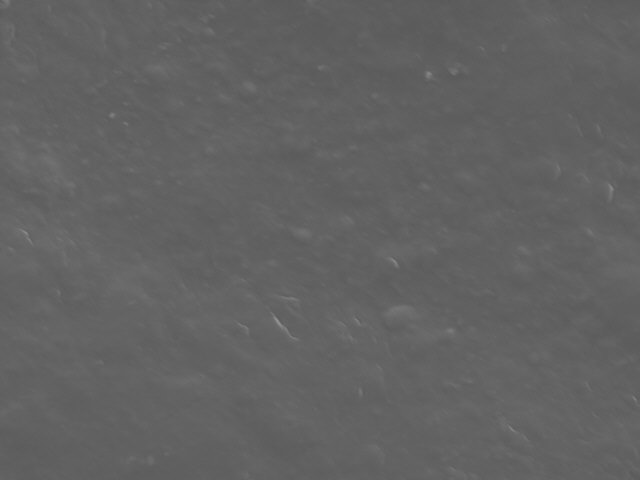

Supplement: S1 File — (ZIP) [file pone.0340257.s005.zip › S1 File/SEM images/KS.tif]

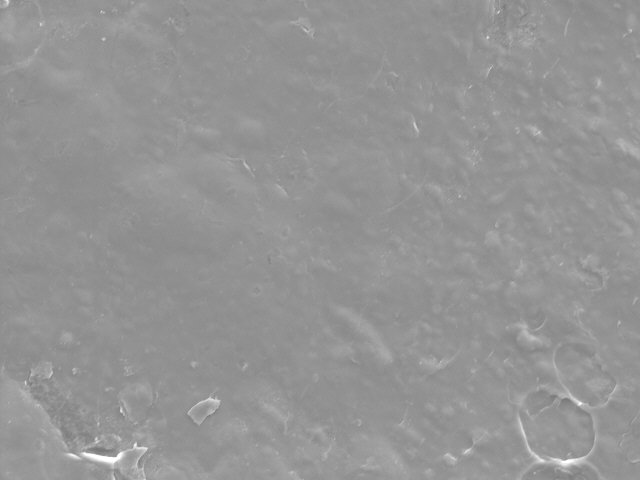

Supplement: S1 File — (ZIP) [file pone.0340257.s005.zip › S1 File/SEM images/KSL5.tif]

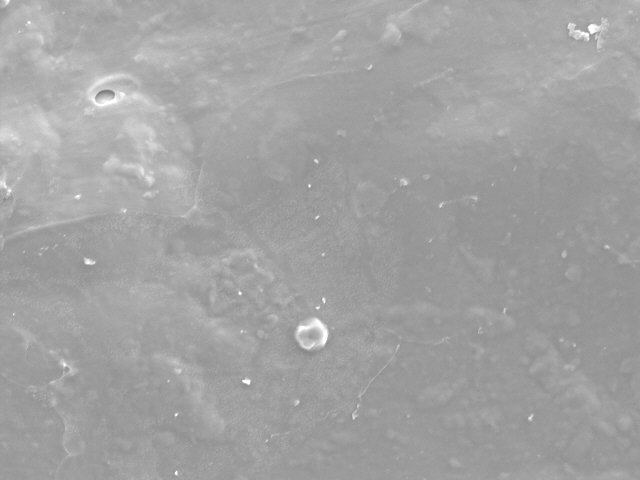

Supplement: S1 File — (ZIP) [file pone.0340257.s005.zip › S1 File/SEM images/KSL6.tif]

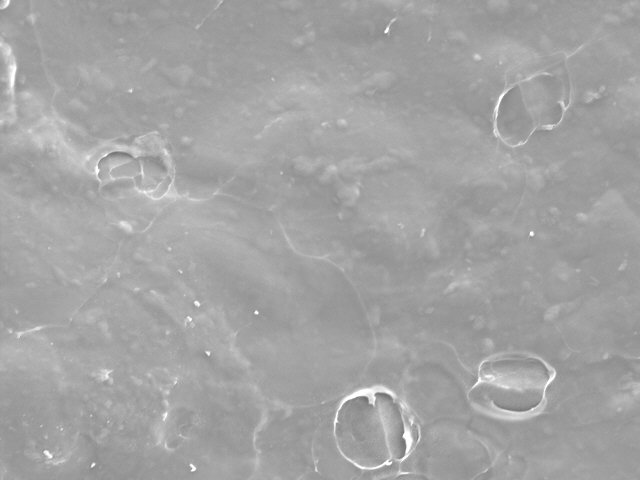

Supplement: S1 File — (ZIP) [file pone.0340257.s005.zip › S1 File/SEM images/KSL7.tif]

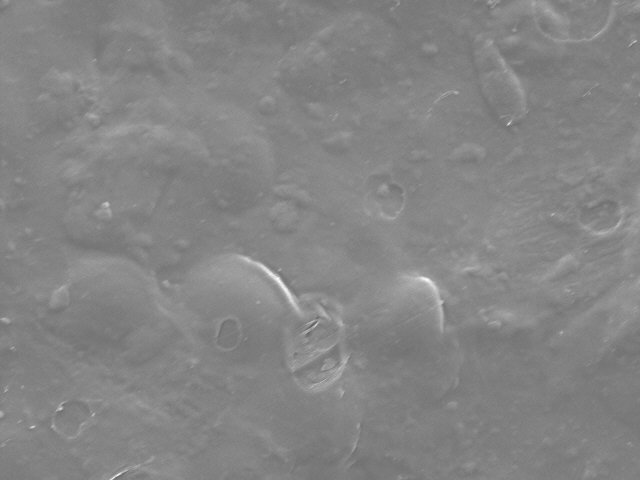

Supplement: S1 File — (ZIP) [file pone.0340257.s005.zip › S1 File/SEM images/KSL8.tif]

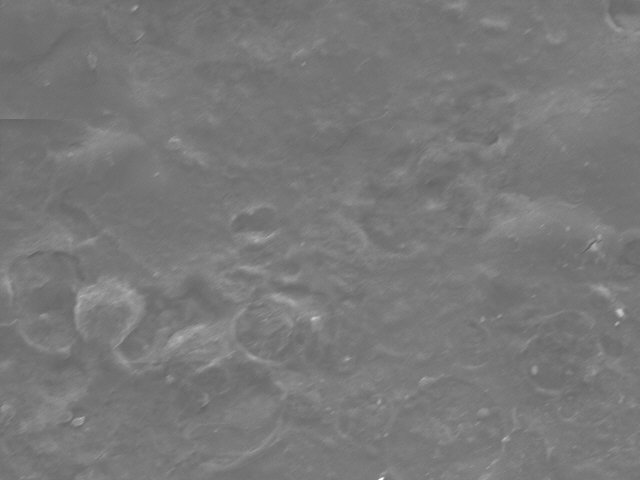

Supplement: S1 File — (ZIP) [file pone.0340257.s005.zip › S1 File/SEM images/KSL9.tif]

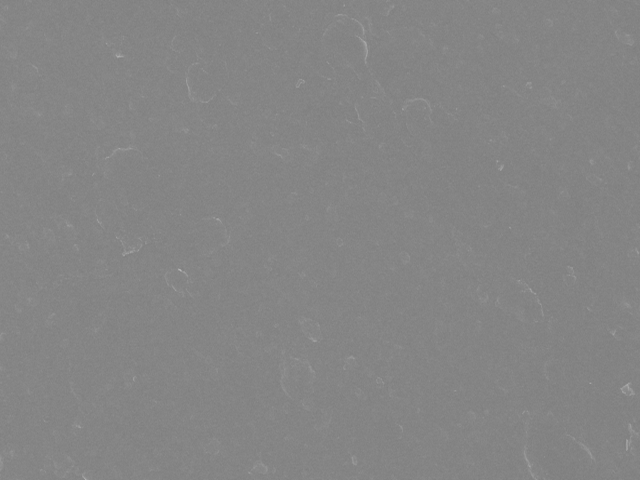

Supplement: S1 File — (ZIP) [file pone.0340257.s005.zip › S1 File/SEM images/KSS10.tif]

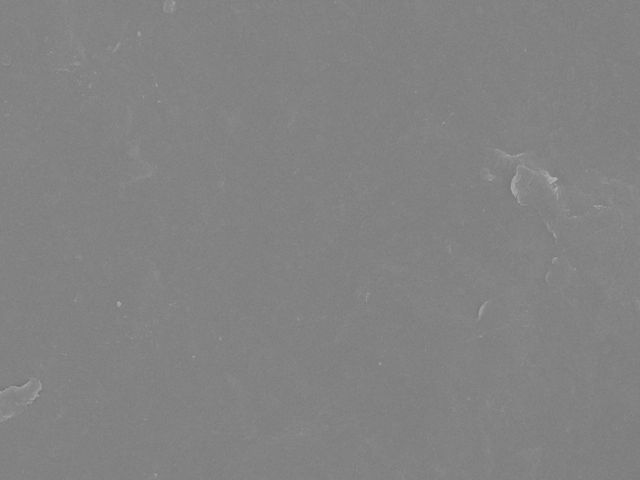

Supplement: S1 File — (ZIP) [file pone.0340257.s005.zip › S1 File/SEM images/KSS2.tif]

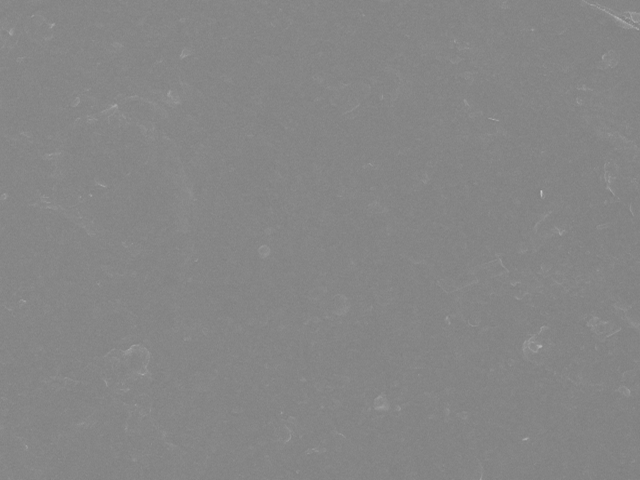

Supplement: S1 File — (ZIP) [file pone.0340257.s005.zip › S1 File/SEM images/KSS4.tif]

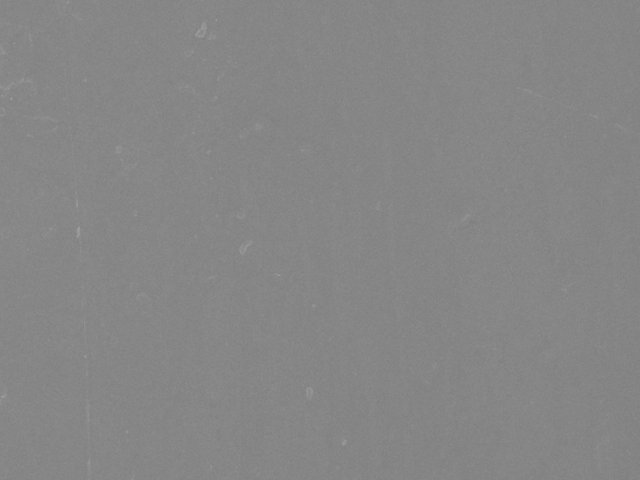

Supplement: S1 File — (ZIP) [file pone.0340257.s005.zip › S1 File/SEM images/KSS6.tif]

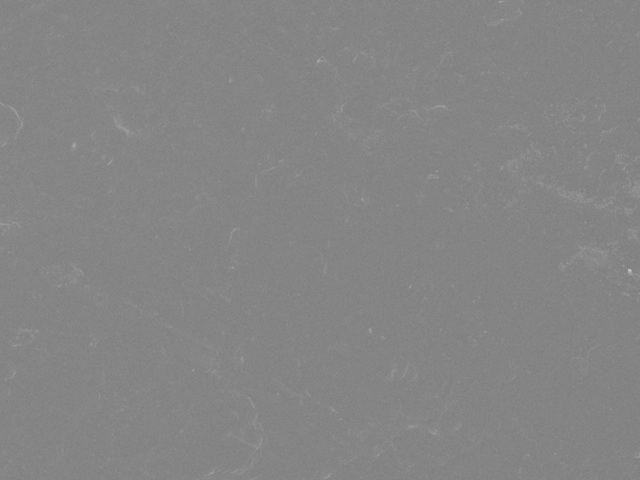

Supplement: S1 File — (ZIP) [file pone.0340257.s005.zip › S1 File/SEM images/KSS8.tif]

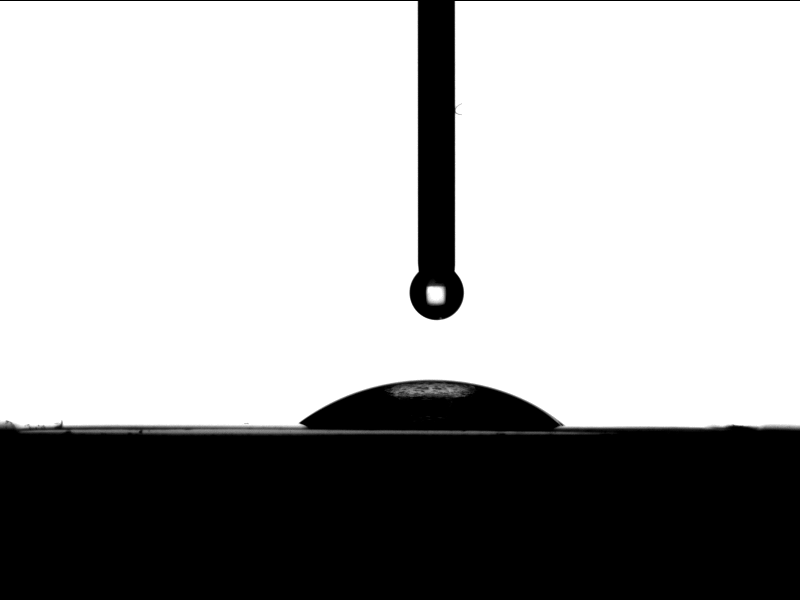

Supplement: S1 File — (ZIP) [file pone.0340257.s005.zip › S1 File/WCA images/KGM.bmp]

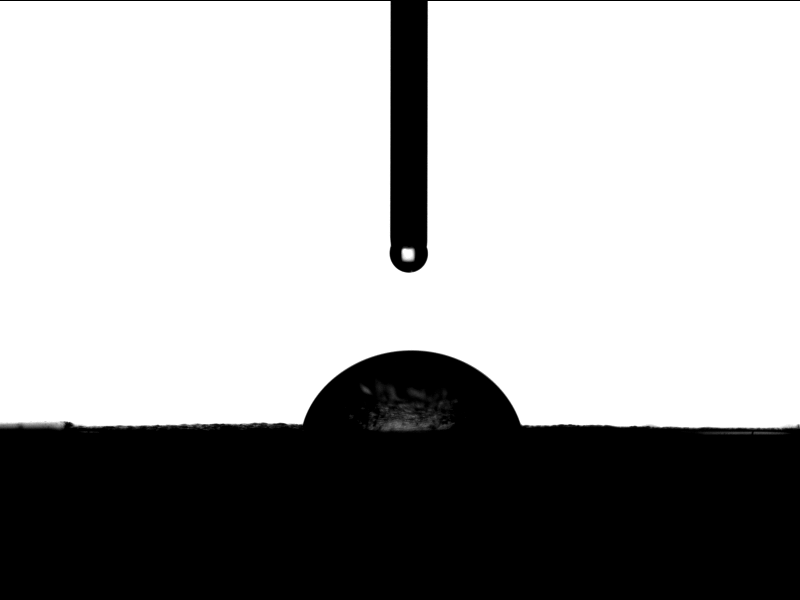

Supplement: S1 File — (ZIP) [file pone.0340257.s005.zip › S1 File/WCA images/KS.bmp]

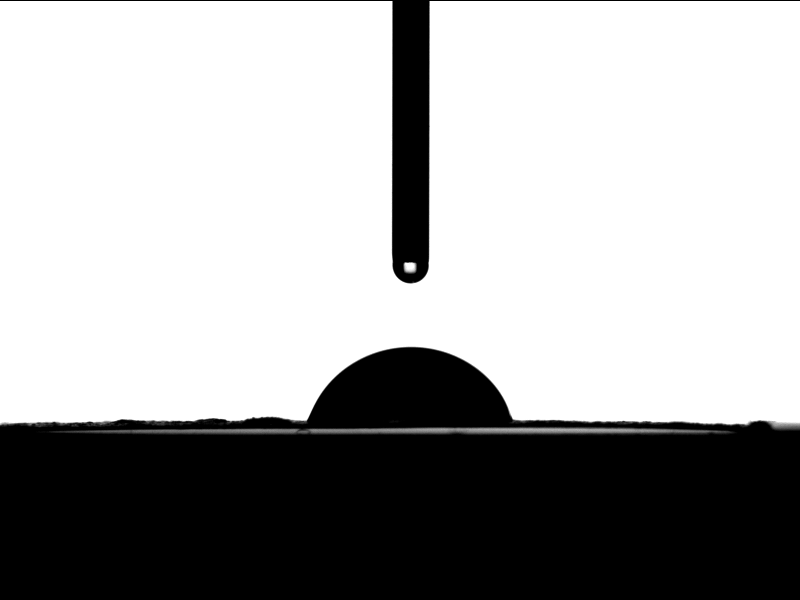

Supplement: S1 File — (ZIP) [file pone.0340257.s005.zip › S1 File/WCA images/KSL5.bmp]

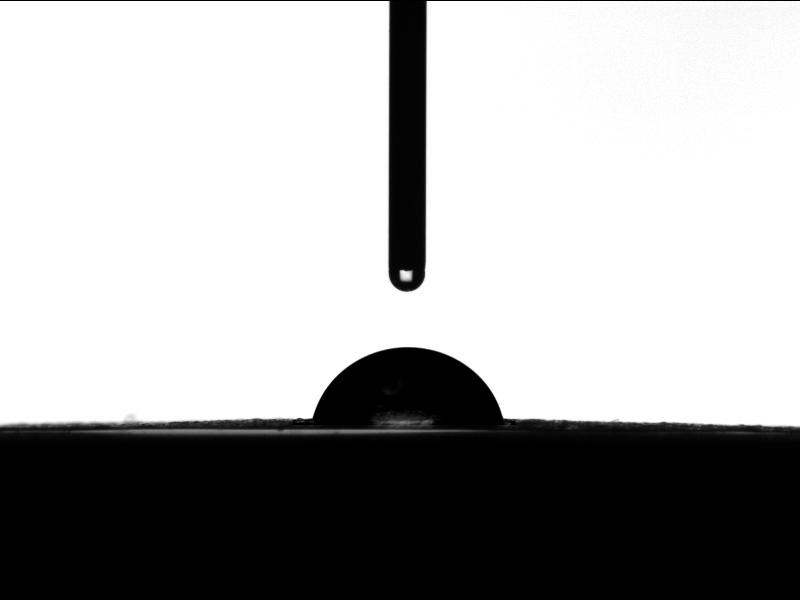

Supplement: S1 File — (ZIP) [file pone.0340257.s005.zip › S1 File/WCA images/KSL6.bmp]

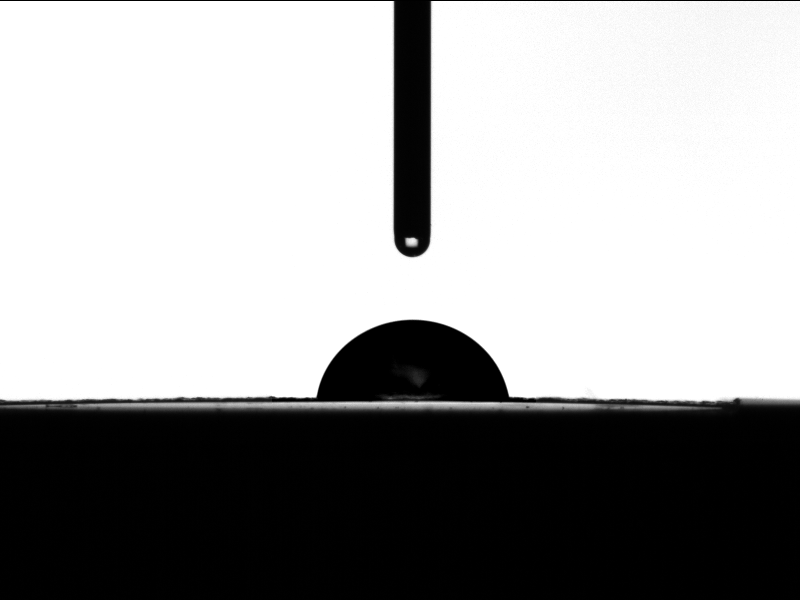

Supplement: S1 File — (ZIP) [file pone.0340257.s005.zip › S1 File/WCA images/KSL7.bmp]

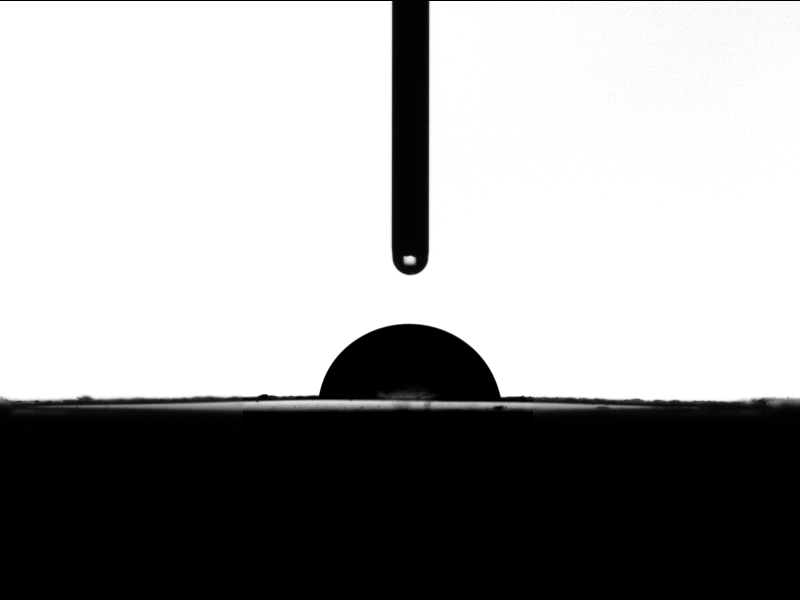

Supplement: S1 File — (ZIP) [file pone.0340257.s005.zip › S1 File/WCA images/KSL8.bmp]

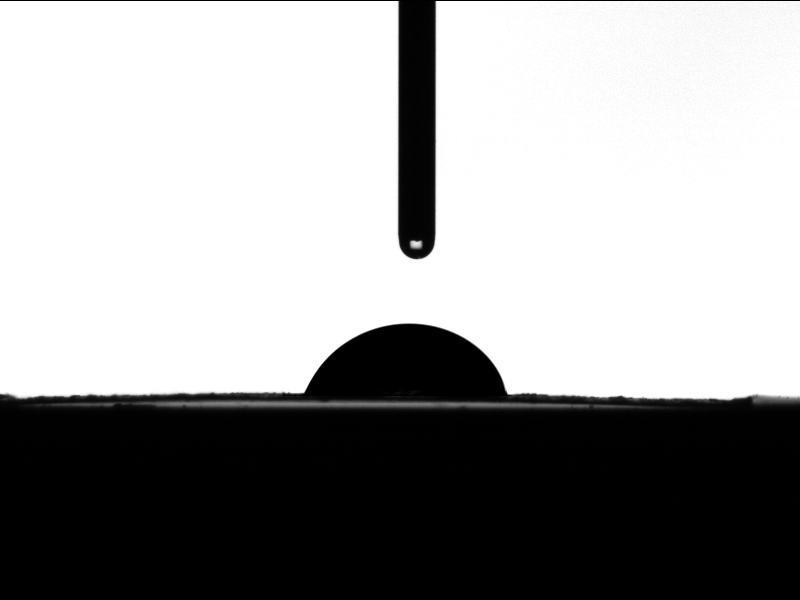

Supplement: S1 File — (ZIP) [file pone.0340257.s005.zip › S1 File/WCA images/KSL9.bmp]

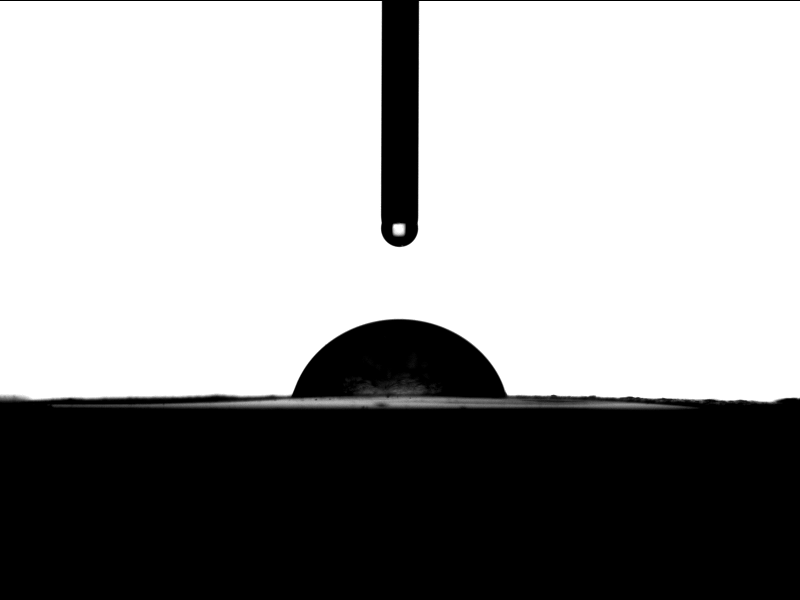

Supplement: S1 File — (ZIP) [file pone.0340257.s005.zip › S1 File/WCA images/KSS10.bmp]

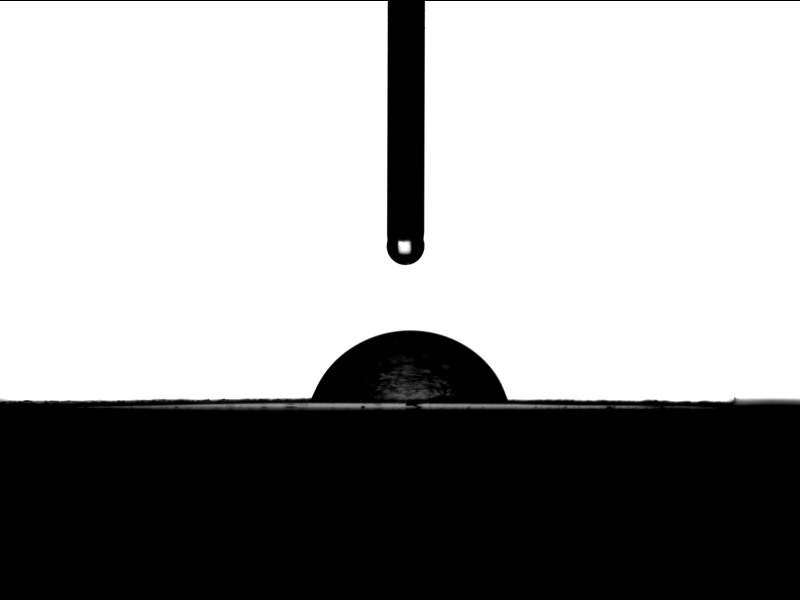

Supplement: S1 File — (ZIP) [file pone.0340257.s005.zip › S1 File/WCA images/KSS2.bmp]

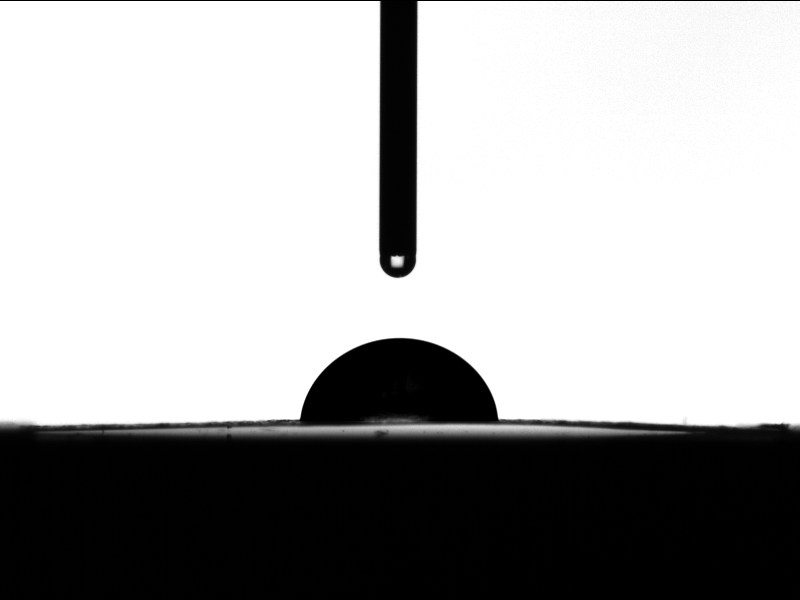

Supplement: S1 File — (ZIP) [file pone.0340257.s005.zip › S1 File/WCA images/KSS4.bmp]

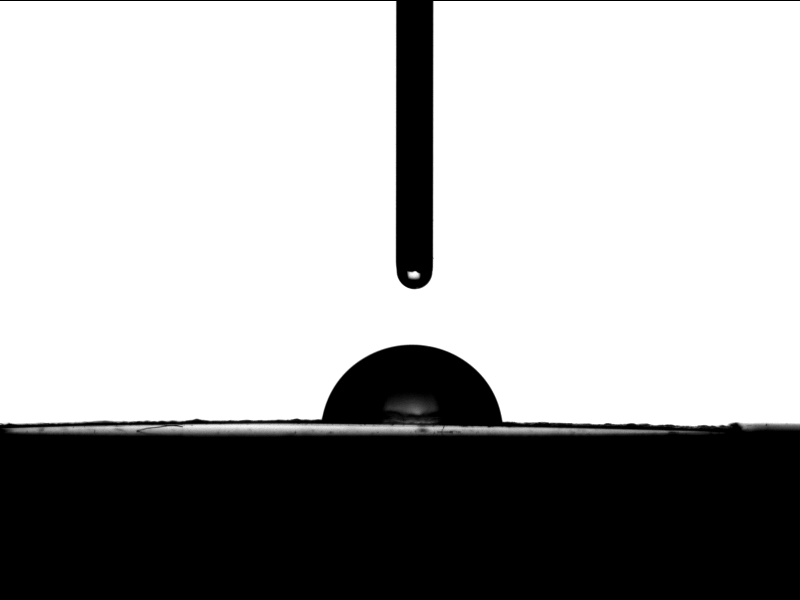

Supplement: S1 File — (ZIP) [file pone.0340257.s005.zip › S1 File/WCA images/KSS6.bmp]

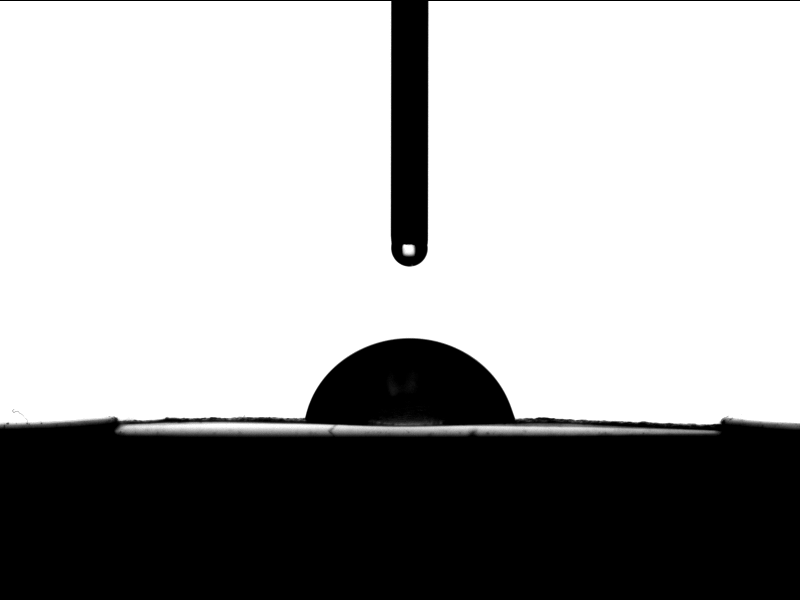

Supplement: S1 File — (ZIP) [file pone.0340257.s005.zip › S1 File/WCA images/KSS8.bmp]

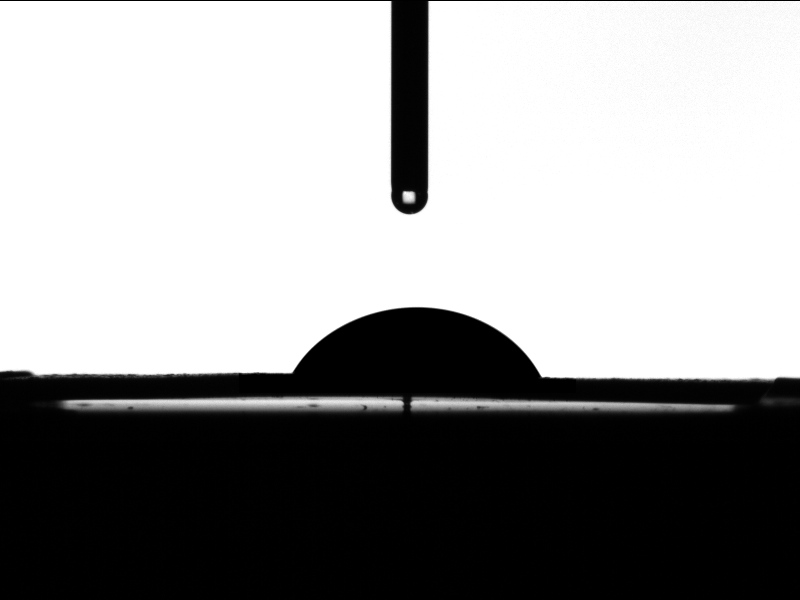

Supplement: S1 File — (ZIP) [file pone.0340257.s005.zip › S1 File/WCA images/SPI.bmp]
